# Comprehensive management of obstructive sleep apnea by telemedicine: Clinical improvement and cost-effectiveness of a Virtual Sleep Unit. A randomized controlled trial

**DOI:** 10.1371/journal.pone.0224069

**Published:** 2019-10-24

**Authors:** Vera M. Lugo, Onintza Garmendia, Monique Suarez-Girón, Marta Torres, Francisco J. Vázquez-Polo, Miguel A. Negrín, Anabel Moraleda, Mariana Roman, Marta Puig, Concepcion Ruiz, Carlos Egea, Juan F. Masa, Ramon Farré, Josep M. Montserrat

**Affiliations:** 1 Sleep Unit, Respiratory Department, Hospital Clínic, Barcelona, Spain; 2 Centro de Investigación Biomédica en Red (CIBER) Enfermedades Respiratorias, Madrid, Spain; 3 Biophysics and Bioengineering Department, School of Medicine and Health Sciences, University of Barcelona, Barcelona, Spain; 4 Institut d’Investigacions Biomèdiques Pi i Sunyer (IDIBAPS), Barcelona, Spain; 5 Quantitative Methods Department and TiDES Institute, Las Palmas de Gran Canaria University, Las Palmas de Gran Canaria, Spain; 6 Sleep Unit, Respiratory Department, Alava University Hospital, Vitoria, Spain; 7 Respiratory Unit, San Pedro de Alcántara Hospital, Cáceres, Spain; 8 Instituto Universitario de investigación Biosanitaria de Extremadura, Cáceres, Spain; 9 Medicine Department, School of Medicine and Health Sciences, University of Barcelona, Barcelona, Spain; Charité - Universitätsmedizin Berlin, GERMANY

## Abstract

**Introduction:**

Obstructive sleep apnea (OSA) is a prevalent disease associated with significant morbidity and high healthcare costs. Information and communication technology could offer cost-effective management options.

**Objectives:**

To evaluate an out-of-hospital Virtual Sleep Unit (VSU) based on telemedicine to manage all patients with suspected OSA, including those with and without continuous positive airway pressure (CPAP) therapy.

**Methods:**

This was an open randomized controlled trial. Patients with suspected OSA were randomized to hospital routine (HR) or VSU groups to compare the clinical improvement and cost-effectiveness in a non-inferiority analysis. Improvement was assessed by changes in the Quebec Sleep Questionnaire (QSQ), EuroQol (EQ-5D and EQ-VAS), and Epworth Sleepiness Scale (ESS). The follow-up was 3 months. Cost-effectiveness was assessed by a Bayesian analysis based on quality-adjusted life-years (QALYs).

**Results:**

The HR group (n: 92; 78% OSA, 57% CPAP) compared with the VSU group (n: 94; 83% OSA, 43% CPAP) showed: CPAP compliance was similar in both groups, the QSQ social interactions domain improved significantly more in the HR group whereas the EQ-VAS improved more in the VSU group. Total and OSA-related costs were lower in the VSU group than the HR. The Bayesian cost-effectiveness analysis showed that VSU was cost-effective for a wide range of willingness to pay for QALYs.

**Conclusions:**

The VSU offered a cost-effective means of improving QALYs than HR. However, the assessment of its clinical improvement was influenced by the choice of the questionnaire; hence, additional measurements of clinical improvement are needed. Our findings indicate that VSU could help with the management of many patients, irrespective of CPAP use.

## Introduction

Obstructive sleep apnea (OSA) is the most common sleep-related breathing disorder, affecting 10%–15% of the general population. It is characterized by repeated episodes of upper airway collapse during sleep that lead to nocturnal hypoxemia and sleep fragmentation [1]. If untreated, OSA is associated with a poorer quality of life and an increased risk for cardiovascular, cerebrovascular, and metabolic diseases as well as traffic accidents [2]. Consequently, patients with OSA pose a significant burden on healthcare resources [3,4].

Greater awareness of sleep disorders, especially OSA, in professional areas and social media over recent years have meant that the demands for sleep care have increased exponentially. However, the response by healthcare services has been inadequate, leading to saturated sleep units with long waiting times, especially in tertiary care. Given this situation, a major change is urgently needed in the way we approach sleep medicine. Given that OSA is such a prevalent and chronic disease, all care levels must be involved in its management [5,6], and there is a need for patient contact, interaction, and management to change. This could be achieved by exploiting the advantages offered by information and communication technologies (ICTs) [7–12]. Such technologies could help to reduce the burden on care providers, offer patients closer monitoring without the need to attend hospital, and reduce health costs. Telemedicine could be a particularly good option for providing personalized and cost-effective health care.

Several studies have tried to demonstrate the feasibility or cost-effectiveness of telemedicine in the management of OSA, but often with contradictory results due partly to their limitations. First, they are typically limited to the follow-up of patients on continuous positive airway pressure (CPAP) and only focus on treatment compliance [12–14]. These fail to consider other aspects of OSA management, such as diagnosis, titration if needed, and follow-up (with or without CPAP). Indeed, it is equally important to monitor and support patients not receiving CPAP, including those following sleep hygiene, diet, or exercise, as well as those being treated for important comorbidities associated to OSA, such as hypertension or diabetes. Second, a number of these telematics studies have compared classical in-hospital management and follow-up with home procedures [11–14], such as in-hospital diagnosis only by polysomnography (PSG) and home diagnosis by home respiratory polygraphy (HRP) [15–17]. However, in reality, both hospital-based and home-based respiratory polygraphy are accepted and integrated in routine hospital care, with up to 60% of sleep tests performed at home. This fact should be considered in cost-effectiveness analyses. Third, most trials have included small samples or have been pilot studies exploring the performance of ICTs devices [18–20].

Useful and effective telematic management of OSA should ideally cover all aspects, including diagnosis, titration if needed and follow-up of CPAP and non-CPAP treatments. We therefore present a new telematic management protocol for OSA based on a Virtual Sleep Unit (VSU) to diagnose, titrate if required, and follow-up all patients with suspected OSA in a home setting.

In this study, we compare the clinical improvement and cost-effectiveness as well as side effects, patient satisfaction and CPAP compliance of the VSU model with routine conventional hospital care including both in-hospital and home tests.

## Methods

This is a prospective, open, randomized study where participants who completed the baseline visit were randomly assigned (1:1) to the control or telemedicine group. Randomization was at an individual level without restriction (i.e., no blocking) and was completely automated by use of an unseen random number function embedded in the data collection website code. This trial was conducted in the Hospital Clinic, Barcelona, where it was approved by the relevant Ethics Committee and registered at ClinicalTrials.gov (NCT02779894). Consecutive patients with suspected OSA referred to our Sleep Unit were enrolled between 2016 and February 2017 and randomized to either hospital routine (HR) or VSU management if they signed an informed consent form.

The inclusion criteria were as follows: suspected OSA and/or refractory hypertension, age 18–75 years, basic knowledge of ICTs use (e.g., tablet, smartphone, or computer), and Internet access. We excluded patients with disabilities that prevented them from completing the questionnaires, invalidating somnolence (medical criteria), unstable disease, previous CPAP use, uvulopalatopharyngoplasty, risk profession or not signing the informed consent form.

### Hospital routine (HR)

Sleep tests, medical assessments, and follow-up visits were performed in the Sleep Unit following our usual procedures. Based on the patient characteristics, physicians not involved in the trial requested sleep studies (e.g. PSG, or hospital- or home-based respiratory polygraphy) as detailed in the online supplementary material (S1 Text). After sleep testing, a sleep physician interviewed patients. If CPAP was indicated, patients received education and training in CPAP use from a specialized nurse or technician in the hospital. CPAP was then titrated in the hospital with manual adjustment by the technician during a sleep study (see online supplementary material S1 Text). Once the optimal pressure was determined, patients were provided with a fixed pressure CPAP device to use at home (DreamStation, Respironics). All visits were performed face-to-face in the consultation at 3, 6 and 12 weeks.

### Virtual Sleep Unit (VSU)

Patients in the VSU were managed exclusively outside of the hospital setting. The diagnostic sleep test consisted of home-based respiratory polygraphy for three consecutive nights, which we recently proved to be as effective as PSG in all patients, using ApneaLink air (ResMed, Spain) [17]. Patients collected and returned the equipment at an external office linked to their healthcare providers. Recorded data were downloaded to a secure server and analyzed by a specialized technician. Subsequently, a sleep physician assessed all records and scheduled a videoconference visit to inform the results and discuss the therapeutic decision.

If OSA was diagnosed and CPAP was indicated, patients received CPAP education and along with an automatic CPAP device (Dreamstation, Respironics) at the providers pick-up point. A technician could remotely adjust CPAP pressure through a website (EncoreAnywhere, Respironics), based on data sent by the device (i.e., pressure, leaks, residual apnoea–hypopnea index, hours of use). Optimal CPAP pressure was determined with the automated device (remote control) during the first days of the treatment and was later maintained at ±3 cmH_2_O, which could be changed as needed during follow-up. Thus, patients were managed remotely and treatment could accurately be controlled (see online supplementary material S1 Text). The time of the interview with the physician was similar between the two arms (no more than 15 minute). Follow-up visits at 3, 6 and 12 weeks were performed through a custom web application (https://plataforma.laboratori-virtual-son.com) developed for the study, with separated areas for patients and professionals or phone-calls. Patients could access general information about OSA, CPAP, healthy sleep, and lifestyle, as well as their medical agenda, FAQs, and online clinical questionnaires. An email address to contact professionals and a teleconference service to perform the interviews were also available. Professionals could schedule and perform teleconference visits, send messages to patients, and analyze the questionnaire responses.

### Interventions and assessments

Before the HR or VSU diagnostic procedures, patients underwent clinical evaluation of anthropometric data, medical history, OSA symptoms, and treatments received. Sleepiness was assessed by the Epworth Sleepiness Scale (ESS) and quality of life by the Quebec Sleep Questionnaire (QSQ) and EuroQol (EQ-5D tariffs and EQ-VAS) [21–23]. Patients were or not diagnosed with OSA and, according to the medical opinions, received CPAP treatment and/or sleep hygiene measures and lifestyle recommendations (e.g., diet, exercise, regular sleep hours, sleeping on their side). Patients receiving CPAP treatment were monitored by a specialized nurse at 3, 6 and 12 weeks (face-to-face or videoconference, according to the study group) to assess their general symptoms, CPAP compliance, and side effects.

At the final visit, all patients, including those who received sleep hygiene and lifestyle advice, were visited to assess the ESS, QSQ, EuroQol, and their overall satisfaction with the diagnostic and treatment procedure. Patients managed by the VSU also answered questions related to specific aspects of the telematic procedure, including the website information, online questionnaires, and teleconference quality.

### Statistics

Statistical analysis was performed with intention-to-treat (ITT) population (all randomized patients) and per protocol (PP) population (patients who finished the study). Sample size was calculated assuming that 85% patients randomized would meet the definition of a PP sample for a non-inferiority test. Considering a one-sided type I error of 0.025 and 80% power to verify that average score in the emotions domain of QSQ in the VSU group was not less than 1 (standard deviation, SD = 2.5) [24], based on a non-inferiority margin of -2 and a 15% drop-out rate, sample size was 92 patients (HR) and 94 (VSU).

Continuous variables are represented by mean±SD and were compared by t-test or non-parametric Mann-Whitney test, as appropriate. Categorical variables are presented as number of patients (percentage) and were compared using Chi-square or Fisher’s exact test. Overall efficacy and non-inferiority analyses were performed with PP sample, comparing VSU with HR by a one-sided 97.5% CI for the point estimate of the difference between groups and calculated by improvement in QSQ, using two-sample t-tests, according to International Committee for Harmonization E9 guidelines [25]. Continuous efficacy measures were compared by ANCOVA adjusted by age, sex and Apnea hypopnea index (AHI). Analyses were conducted using Stata Release 15.

### Cost analysis

The cost analysis, described in detail in online supplementary material (S2 Text), was performed considering total costs and OSA-related costs, and including direct medical costs (healthcare resources: sleep studies, material replacements, staff salaries and pharmaceutical consumption), direct non-medical costs (travel expenses incurred by patients) and indirect costs (lost productivity due to medical visits) [12]. Indirect costs were estimated based on the average salary in Spain adjusted by gender and educational level [26]. The number of extra visits (general practitioners, specialists, hospital admissions, emergency and intensive care unit visits) was also recorded, and their unit costs were provided by the administrative department of the hospital.

### Cost-effectiveness analysis

Cost-effectiveness analysis, which is detailed in the supplementary material (S3 Text), was carried out from a Bayesian perspective to permit a more robust estimation of the precision of the estimates, and a more natural interpretation of the uncertainty of the results in terms of probability [12,27]. It was assessed considering Quality-adjusted life years (QALYs), which were estimated from the EQ-5D tariffs [28]. Cost-effectiveness was also analyzed considering QSQ, which was one of the main outcomes of the trial. All cost-effectiveness analyses were conducted using OpenBUGS [29].

## Results

**Fig 1:**
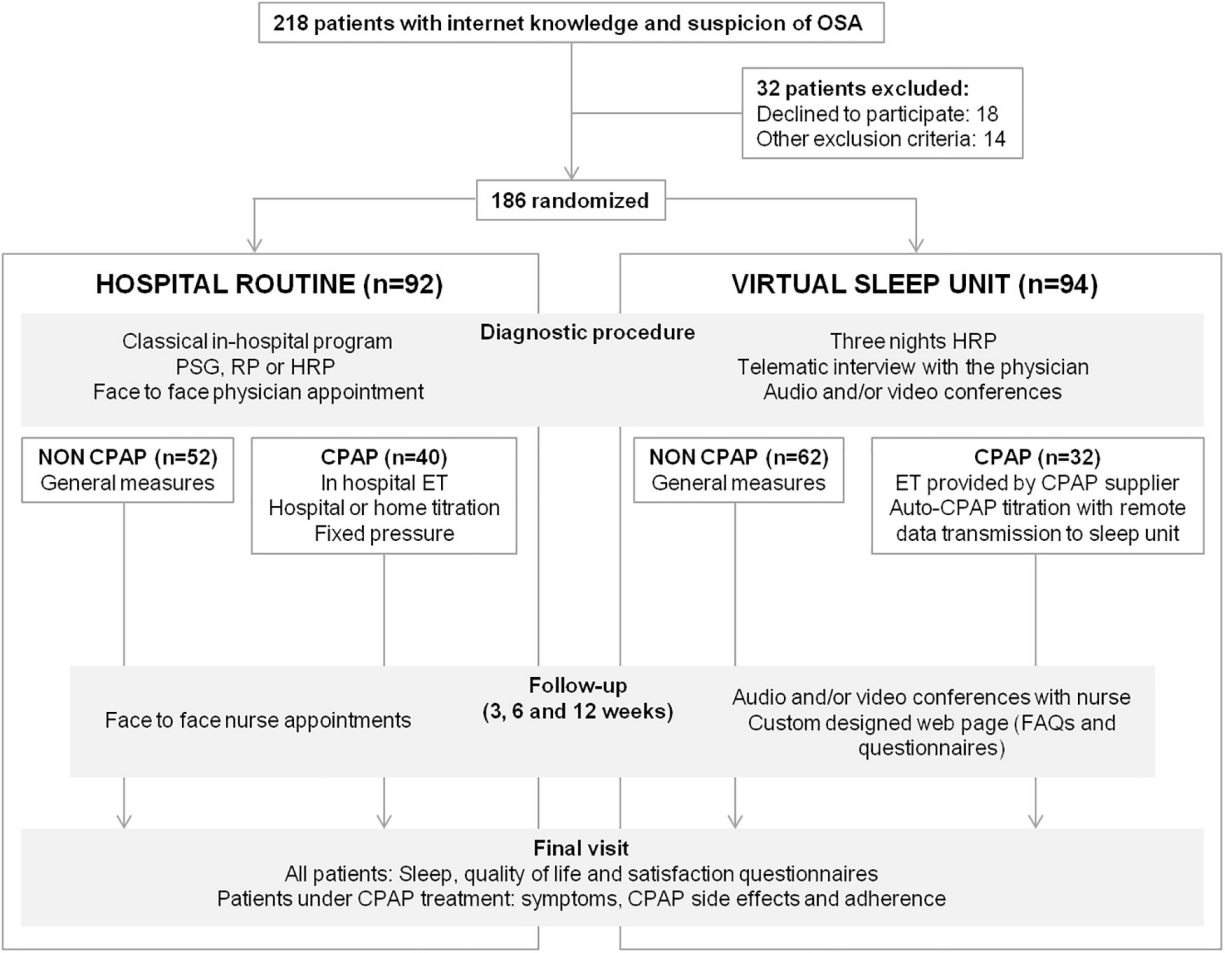
Study flowchart. Protocol flowchart. ET: educational training. FAQs: frequent asked questions. HRP: home respiratory polygraphy. PSG: full polysomnography. RP: respiratory polygraphy.

Two hundred eighteen patients were screened to participate in the trial, of which 32 were excluded: 18 declined to participate and 14 did not meet all the inclusion or exclusion criteria (Fig 1). Thus, 186 patients (ITT population) were randomized to either HR or VSU, and 154 (83%) completed all visits and tests (PP population). As shown in Table 1, ITT population was aged 50.6±11.7 years and 68% were male patients, with an average BMI of 30.73±8.85 kg/m^2^. Baseline characteristics were similar in both groups of randomization, with the exception of a lower neck circumference and a higher incidence of alcohol consumption and nasal obstruction in the VSU group.

**Table 1:** Baseline characteristics. ITT Population.

	All patients (n = 186)	Virtual Sleep Unit (n = 94)	Hospital routine (n = 92)
Male gender	127 (68.3)	66 (70.2)	61 (66.3)
Mean age (years)zz	50.60 ± 11.70	50.39 ± 11.31	50.82 ± 12.15
Neck circumference (cm)	40.00 ± 4.74	38.99 ± 3.97	41.03 ± 5.24
BMI (Kg/m^2^)	30.73 ± 8.85	29.97 ± 6.19	31.50 ± 10.91
Nasal obstruction	87 (46.8)	51 (54.3)	36 (39.1)
Smokers	46 (24.7)	21 (22.3)	25 (27.2)
Alcohol users	124 (66.7)	74 (78.7)	50 (54.3)
*Hypertension*	72 (38.7)	38 (40.4)	34 (37.0)
*Diabetes mellitus*	26 (14)	11 (11.7)	15 (16.3)
*Dislipidemia*	101 (54.3)	48 (51.1)	53 (57.6)
*Cardiovascular disease*	19 (10.3)	9 (9.5)	10 (10.9)
*Neurological disease*	12 (6.5)	4 (4.3)	8 (8.7)
*Respiratory disease*	23 (12.4)	11 (11.7)	12 (13)
*Depression*	32 (17.2)	17 (18.1)	15 (16.3)
*Anxiety*	33 (17.7)	20(21.3)	13(14.1)
*Cancer*	16 (8.6)	9 (9.6)	7 (7.6)
OSA diagnosis	144 (80.4)	74 (83.1)	70 (77.8)
CPAP indication	72 (50.0)	32 (43.2)	40 (57.1)
AHI	29.12 ± 25.60	24.68 ± 21.01	33.60 ± 28.96
ODI3%	28.50 ± 24.23	24.18 ± 20.45	32.73 ± 26.88
CT90%	14.37 ± 18.85	15.46 ± 19.38	13.27 ± 18.35
QSQ*	25.82 ± 5.00	25.95 ± 5.27	25.69 ± 4.73
EuroQol-5D	0.81 ± 0.19	0.80 ± 0.19	0.82 ± 0.19
EuroQol-VAS	74.55 ± 54.56	77.98 ± 74.39	71.05 ± 19.29
ESS	9.94 ± 4.76	9.95 ± 4.36	9.92 ± 5.17

Data are expressed by number of patients (%) or mean ± SD. OSA is defined by AHI>10. AHI: apnea hypopnea index. BMI: body mass index. CT90%: percentage of time with oxygen saturation <90%. CPAP: continuous positive airway pressure. ESS: Epworth sleepiness scale. ITT: intention to treat. ODI3%: oxygen desaturation index of 3%. OSA: obstructive sleep apnea. QSQ: Quebec Sleep Questionnaire.

*See basal QSQ domains in Table 2

### Clinical assessments

As described in Table 1, the same proportion of OSA diagnosis was made in both randomization groups: after the sleep test, 83% of patients were diagnosed with OSA in the VSU, while 78% following the HR. The amount of CPAP prescription was 43% in the VSU versus 57% in the HR. This increased number of CPAP indication in HR was not statistically significant. In addition, there were no differences in sleep test parameters or initial clinical questionnaires. Baseline differences between patients with or without CPAP treatment are detailed in S1 Table in online supplementary material.

Table 2 shows the changes in the clinical questionnaires after follow-up. Total score of QSQ was similar in both groups of randomization. When QSQ domains were analyzed, significant differences favoring the HR were found in the domain referring to social interactions (-0.480; 95%CI; -0.844 to -0.116; p = 0.010). Conversely, quality of life measured by the EuroQol showed an improvement in the patients managed in the VSU, especially in EQ-VAS (5.578; 95%CI; -0.113 to 11.044; p = 0.046).

**Table 2:** Quality of life and sleepiness questionnaires of PP population. Comparison of Virtual Sleep Unit and Hospital routine.

	Virtual Sleep Unit (n = 80)		Hospital routine (n = 74)		LS mean difference (Virtual Sleep Unit minus Hospital routine)	95% CI for the difference		
	Baseline	Follow-up	Baseline	Follow-up		Lower limit	Upper limit	p value
QSQ	25.77 ± 5.34	27.28 ± 5.34	26.11 ± 4.63	29.22 ± 4.12	-1,114	-2.338	0.109	0.074
Daytime hypersomnia	5.32 ± 1.30	5.63 ± 1.19	5.61 ± 1.20	6.21 ± 0.85	-0.206	-0.546	0.134	0.233
Diurnal symptoms	4.90 ± 1.39	5.17 ± 1.37	5.17 ± 1.49	5.69 ± 1.16	-0.166	-0.499	0.167	0.326
Nocturnal symptoms	4.71 ± 1.22	5.37 ± 1.18	4.73 ± 1.26	5.61 ± 1.03	-0.051	-0.411	-0.380	0.779
Emotions	5.44 ± 1.17	5.59 ± 1.12	5.51 ± 1.04	5.92 ± 1.03	-0.211	-0.449	-0.027	0.082
Social interactions	5.40 ± 1.37	5.51 ± 1.23	5.08 ± 1.25	5.78 ± 1.13	-0.480	-0.844	-0.116	**0.010**
EuroQol-5D	0.80 ± 0.18	0.84 ± 18	0.84 ± 0.18	0.85 ± 0.16	0.042	-0.004	0.087	0.074
EuroQol-VAS	70.46 ± 16.84	75.66 ± 13.68	73.70 ± 17.44	75.09 ± 17.35	5.578	-0.113	11.044	**0.046**
ESS	10.36 ± 4.07	8.50 ± 4.44	9.74 ± 5.26	7.05 ± 4.31	0.626	-0.623	1.874	0.324

Data are expressed by mean ± SD. LS mean analysis is based on an ANCOVA model adjusted by age, sex and AHI for change from baseline to follow-up in the questionnaire variables as response to treatment group in per protocol population. ANCOVA: analysis of covariance. BMI: body mass index. CI: confidence interval. ESS: Epworth sleepiness scale. LS mean: least square mean. QSQ: Quebec Sleep Questionnaire. QoL: quality of life. VAS: visual analogue scale.

Regarding sleepiness, the ESS improved significantly more in patients treated with CPAP in the HR group. However, although there were no statistically significant differences in the baseline ESS scores, patients treated in hospital who finally received CPAP had slightly higher ESS scores than their peers in the VSU (S2 and S3 Tables).

The different titration procedures in VSU and HR resulted in similar final CPAP pressure values (8.47±1.61 and 9.29±1.96 respectively, p = 0.120), with no significant difference in mean CPAP compliance: 5.68±1.38 hours in the VSU and 5.63±1.64 in the hospital. Therefore, CPAP use was higher than 5 hours/night, and all CPAP patients reported an improvement in terms of residual sleepiness and general symptoms.

### Cost analysis

Table 3 shows average total costs and OSA-related costs of direct medical, direct non-medical and indirect costs. When comparing VSU and the HR, no differences were found in direct medical costs, which are the main component of the total costs for both groups (91.97% for the VSU and 87.18% for the HR). However, direct medical costs were lower in the VSU than the HR when considering only OSA-related costs (233.06±267.49 € and 319.96±290.75 €, p<0.0001). The VSU also showed lower direct non-medical costs than the HR, for both total and OSA-related costs (p<0.0001), while indirect costs were similar in both VSU and HR. Average total costs were estimated in 494.14 € per person for the VSU and 632.52 € per person for the HR (p<0.032). When OSA-related costs were considered, the VSU also showed lower costs than the HR (257.53 € and 390.96 €, p<0.0001, respectively). Although costs for patients with CPAP were higher, OSA-related costs were lower in the VSU, regardless whether patients were under CPAP treatment or not. Costs were 457.27 € and 628.69 € in the VSU and in HR respectively (p<0.004) for CPAP patients 154.44 € and 208.09 € (p<0.0001) for non-CPAP patients.

**Table 3:** Cost analysis.

	Virtual Sleep Unit	Hospital routine	p value
Direct medical costs	454.45 ± 400.83	551.43 ± 515.20	0.162
Direct medical costs (OSA-related)	233.06 ± 267.49	319.96 ± 290.75	**<0.0001**
Direct non-medical costs	16.13 ± 12.70	28.92 ± 25.54	**<0.0001**
Direct non-medical costs (OSA-related)	14.08 ± 11.89	26.25 ± 20.62	**<0.0001**
Indirect costs	23.56 ± 49.22	52.17 ± 144.89	0.866
Indirect costs (OSA-related)	10.40 ± 24.95	44.74 ± 114.77	0.383
Total costs	494.14 ± 434.11	632.52 ± 574.43	**0.032**
Total costs (OSA-related)	257.53 ± 280.65	390.96 ± 317.31	**<0.0001**

Data are expressed in € by mean ± SD.

The number of total recorded extra visits and the amount of material replacements (mask interfaces, humidifiers and adapters) for the 3 months follow up were similar in both treatment groups and are detailed in the online supplementary material (S4 Table).

### Cost-effectiveness analysis

Table 4 summarizes the cost-effectiveness analysis. The mean total costs, estimated by the Bayesian analysis, were 557.54 € and 710.88 € for the VSU and the HR, respectively. The incremental cost was estimated in -153.34 € and the probability that the VSU would be cheaper than the HR was 93.43%. Similar results were found when considering OSA-related costs (264.96 € and 412.03 € for the VSU and the HR, respectively), since the incremental cost was estimated in -147.07 € and the probability that the VSU would be cheaper than the HR procedures was 99.72%.

**Table 4:** Cost-effectiveness analysis.

	Virtual Sleep Unit		Hospital routine		Difference (Incremental)	
	Mean ± SD	95% CI	Mean ± SD	95% CI	Mean ± SD	95% CI
Total cost (€)	557.54 ± 63.21	(452.0, 699.4)	710.88 ± 85.27	(568.8, 901.7)	-153.34 ± 106.2	(-371.6, 46.40)
OSA-related cost (€)	264.96 ± 28.49	(217.1, 328.8)	412.03 ± 48.77	(330.8, 521.2)	-147.07 ± 56.53	(-267.5, -43.9)
QALYs (units)	0.0115 ± 0.0132	(-0.0145, 0.0376)	0.0007 ± 0.0145	(-0.0280, 0.0295)	0.0108 ± 0.0197	(-0.0279, 0.0497)

Data are expressed by mean ± SD.

Effectiveness was higher in the VSU (mean improvement of 0.0115 units) than in the HR (0.0007 units). Thus, the incremental effectiveness was estimated in 0.0108 QALYs, and the probability that the VSU would be more effective than the HR was 70.93%. Conversely, when measured by mean improvement in QSQ values, effectiveness was lower in VSU patients than in those followed in the HR (1.51 and 3.05 units, respectively) and the incremental effectiveness of the VSU was -1.54.

**Fig 2:**
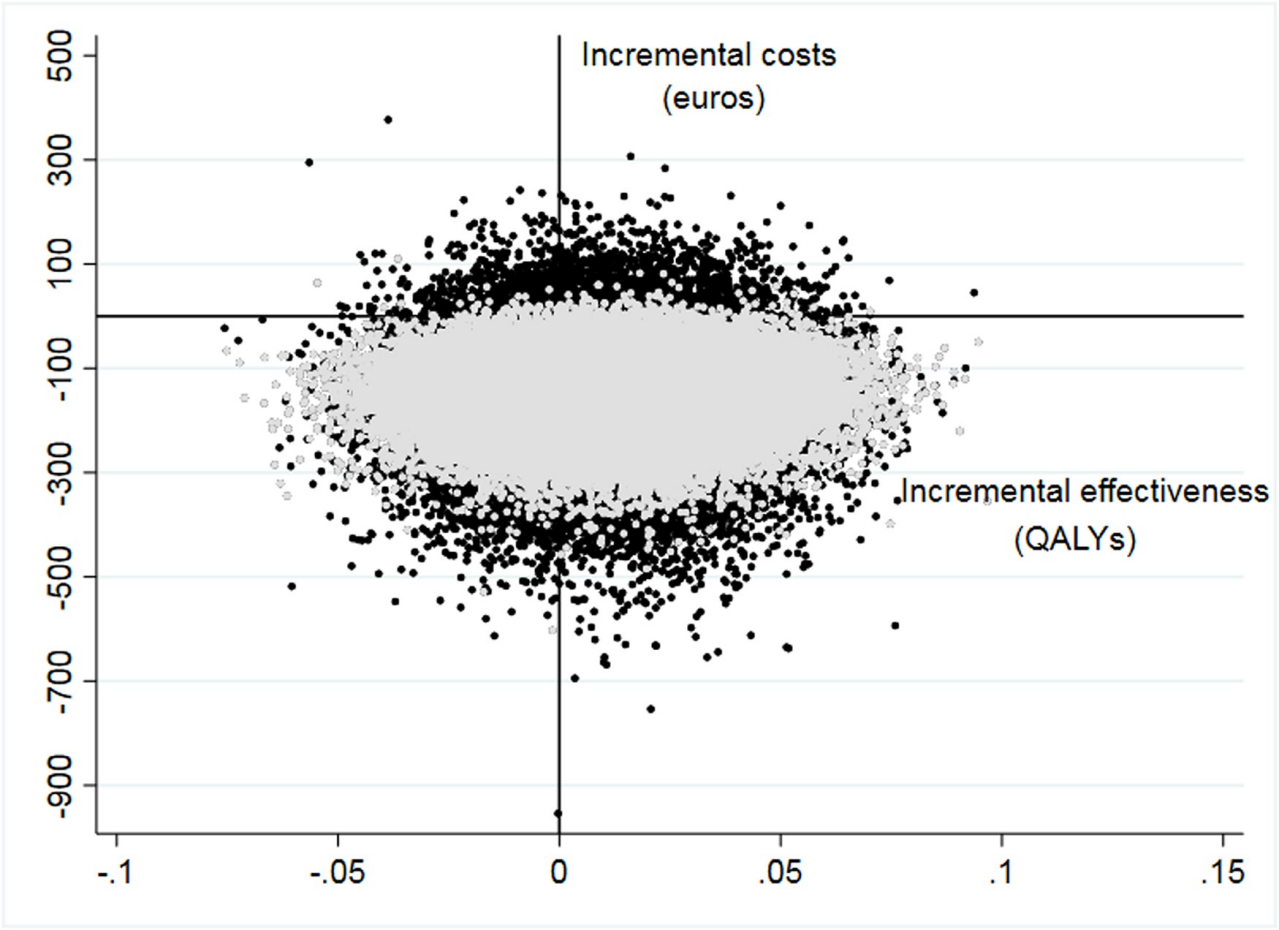
Cost-effectiveness plane. Scatterplot showing the posterior incremental cost and effectiveness measured by QALYs. Grey dots represent OSA-related costs and black dots represent total costs.

**Fig 3:**
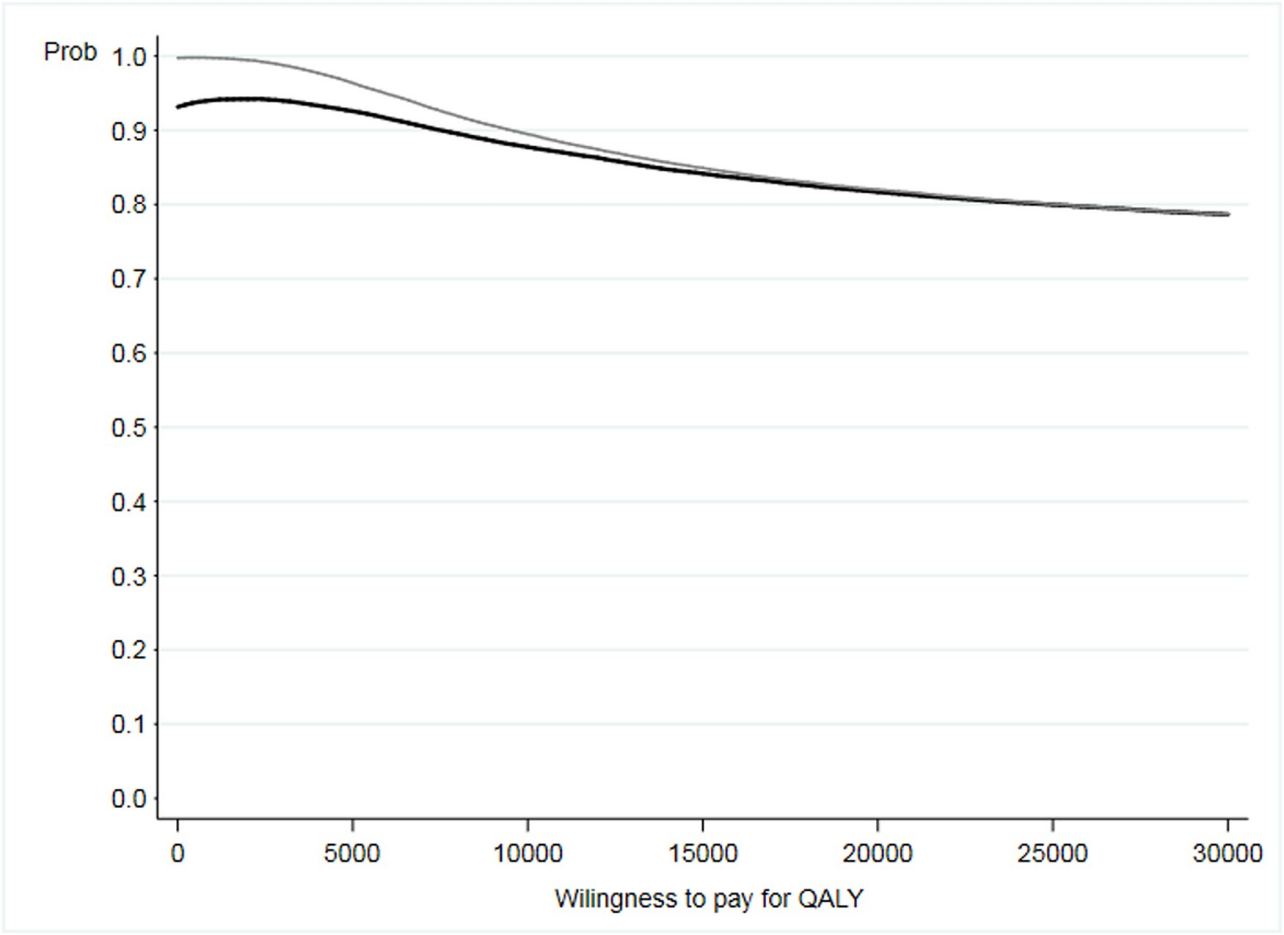
Cost-effectiveness acceptability curve. Grey line represents OSA-related costs and black line represents total costs.

The cost-effectiveness plane (Fig 2) shows the posterior distribution of the incremental effectiveness as well as the incremental cost for total and OSA-related costs. Fig 3 shows the cost-effectiveness acceptability curve (CEAC), which represents the probability of preference for the VSU for a range of willingness to pay for a QALY. The VSU was found to be cost-effective with a probability higher than 78% for the entire range of willingness to pay for a QALY between 0 and 30,000 €.

### Satisfaction survey

The appreciation of the processes at the end follow-up showed that overall patients managed using VSU telemedicine reported a global satisfaction. However, in some items the hospital routine was superior (S5 Table).

## Discussion

We compared the clinical improvement and the cost-effectiveness of a VSU for the management of all patients with suspected OSA against HR care provision in a Spanish sleep unit. To our knowledge this was the first trial to include patients with no CPAP prescription during follow-up and to consider all the procedures involved in HR care, including not only PSG but also hospital- and home-based respiratory polygraphy for diagnostic. The trial results prove the non-inferiority and cost-effectiveness of the VSU, even when considering cheaper hospital- and home-based respiratory polygraphy (compared with PSG) in the HR group.

The 186 participants included in the trial were a much larger sample than the ones previously described in studies on this topic [17–20]. After randomization, the baseline characteristics of patients in the VSU and HR groups were comparable. Although baseline differences in alcohol intake, neck circumference, and nasal obstruction were statistically significant between the two groups, they have no notable clinical relevance and expectedly will not have altered any outcomes. The average age of the participants around 50 years and mostly males, also reflects a typical OSA population.

Overall, 80% of patients were diagnosed with OSA after sleep tests, and of these, CPAP was indicated in 50%. Although not statistically significant, CPAP was prescribed at slightly different rates in the VSU group (43%) and HR group (57%). This could be explained by the fact that home-based respiratory polygraphy underestimates the apnea–hypopnea index and patients with an AHI> 30 tend to be treated more with CPAP. It should be mentioned that the same physician made all diagnoses in both groups. Although this ensured homogeneity of the treatment criteria, it could represent a bias, limiting the generalization of the results to other center.

Conflicting results were obtained when assessing quality of life, consistent with the findings of other studies [12,24,30]. Patients in the HR group showed greater improvement in the social domain of the QSQ, whereas patients in the VSU group showed greater improvements in the EuroQol, particularly the EQ-VAS. This highlights how critical it can be to choose a questionnaire for assessing quality of life, as pointed in a recent systematic review, which focus attention on the need for more research in the study of the properties of patient-reported quality of life measures in adults with OSA [31].

The cost analysis clearly showed that the total costs required for the VSU were lower than those required for HR. Approximately 50% of direct medical costs were unrelated to OSA, with a significant part of the remaining 50% due to the need for extra visits to different health services, and these were similar in both groups. Therefore, the savings associated to the VSU were related to the lower direct medical costs and the travel expenses [32–35], consequently, the VSU could represent an optimal alternative specially if applied to residents in medically underserved areas.

Our results indicate that the costs of the VSU would almost certainly be cheaper than those for HR care, and the Bayesian analysis showed that the VSU was also cost-effective. The cost-effectiveness analysis was also conducted with QSQ, showing inconclusive results. In this analysis the VSU was cheaper but less effective than the HR. If a cost-effectiveness analysis shows no dominance of a treatment, the final decision will depend on how much the decision-maker is willing to pay to increase the effectiveness. Therefore, and since there are no scientific studies that have quantified the willingness to pay for a QSQ unit, there is no data available to evaluate the cost-effectiveness of VSU based on the QSQ. This fact once again highlights the importance of choosing the appropriate questionnaires when assessing quality of life. Compliance with CPAP treatment was high in both groups (> 5 hours) so the impact of VSU on the use of CPAP cannot be established. It is possible that the VSU would be useful in centers with lower compliance rates.

As found in similar studies, patients were satisfied with the telematic diagnostic tools and follow-up procedures and acceptance of ICTs use was high [12,33,34]. This has special relevance in light of the potential implementation of such strategies in usual care, as suggested in recently [10,11,36,37].

On balance, as stated by Scott Wilson and Peter Cram, more time and further knowledge acquisition is still needed before we can implement telemedicine on a larger scale [38]. We have learned that it is necessary, as far as possible, to simplify the processes, always trying to use the simplest and most appropriate tools to meet the patient’s needs, and even agreeing preferred communication methods with the patients. In addition, a good coordination with companies as well as an appropriate training for both patients and professionals is recommended.

## Supporting information

S1 Text Sleep studies. (DOCX)

S2 Text Costs analysis. (DOCX)

S3 Text Cost-effectiveness analysis. (DOCX)

S1 Table Sleep baseline characteristics and questionnaires in non-CPAP and CPAP patients. **ITT Population**. Data are expressed by mean ± SD. AHI: Apnea hipopnea index. CT90%: percentage of time with oxygen saturation <90%. ESS: Epworth sleepiness scale. ODI3%: Oxygen desaturation index 3%. QSQ: Quebec Sleep Questionnaire. QoL: quality of life. VAS: visual analogue scale. *See domains of QSQ for non CPAP and CPAP patients in S2 and S3 Tables.
(DOCX)

S2 Table Quality of life and sleepiness questionnaires of non-CPAP patients. **Comparison of Virtual Sleep Unit and Hospital routine**. Patients without CPAP indication after sleep study from per protocol population. Data are expressed by mean ± SD. LS mean analysis is based on an ANCOVA model adjusted by age, sex and AHI for change from baseline to follow-up in the questionnaire variables as response to treatment group in per protocol population.ANCOVA: analysis of covariance. BMI: body mass index. CI: confidence interval. ESS: Epworth sleepiness scale. LS mean: least square mean. QSQ: Quebec Sleep Questionnaire. QoL: quality of life. VAS: visual analogue scale.
(DOCX)

S3 Table Quality of life and sleepiness questionnaires of patients under CPAP treatment. **Comparison of Virtual Sleep Unit and Hospital routine**. Patients under CPAP treatment after sleep study from per protocol population. Data are expressed by mean ± SD. LS mean analysis is based on an ANCOVA model adjusted by age, sex and AHI for change from baseline to follow-up in the questionnaire variables as response to treatment group in per protocol population. ANCOVA: analysis of covariance. BMI: body mass index. CI: confidence interval. ESS: Epworth sleepiness scale. LS mean: least square mean. QSQ: Quebec Sleep Questionnaire. QoL: quality of life. VAS: visual analogue scale.
(DOCX)

S4 Table Total number of extra visits and total number of CPAP material replacements. (DOCX)

S5 Table Degree of satisfaction. (DOCX)

S1 File CONSORT Checklist. (DOCX)

S2 File Study Protocol (English). (DOCX)

S3 File Study Protocol (Spanish). (PDF)

S4 File Methodology modifications (Spanish). (DOCX)
