# Supplementary material for: Comprehensive management of obstructive sleep apnea by telemedicine: Clinical improvement and cost-effectiveness of a Virtual Sleep Unit. A randomized controlled trial
Source: PLoS One. 2019 Oct 24;14(10):e0224069. doi: 10.1371/journal.pone.0224069 (PMC6812794; doi:10.1371/journal.pone.0224069)
Supplement: S1 Text — (DOCX) [file pone.0224069.s001.docx]

Sleep studies

According to the hospital routine (HR), patients underwent to different sleep studies: in-hospital full polysomnography (PSG), in-hospital respiratory polygraphy (RP) or home respiratory polygraphy (HRP).

PSG was performed using Grael/Somté PSG (Compumedics Limited 2006, Abbotsford, Victoria, Australia). The variables recorded were electroencephalogram (EEG- leads F4-M1, C4-M1 and O2-M1), electrooculogram (EOG) chin and leg electromyogram (EMG), and electrocardiogram (ECG). Respiratory variables were flow (nasal cannula and oronasal thermistor), respiratory effort, and oxygen saturation (SpO2). Body position, snore (tracheal microphone) and video were also monitored.

RP was performed by Somtè PSG (Compumedics Limited 2006, Abbotsford, Victoria, Australia). The variables measured were flow (nasal cannula), respiratory effort, SpO2, body position, snore (tracheal microphone) and video.

HRP was carried out with a portable type 3 ApneaLink air (ResMed, Spain). The registered variables were flow (nasal cannula), respiratory effort, SpO2, pulse frequency, body position and snore.

In the Virtual Sleep Unit (VSU) OSA diagnosis was performed by HRP for 3 consecutive nights (3N-HRP) also using ApneaLink air (ResMed, Spain), as described in Guerrero A et al. Sleep. 2014 Aug 1; 37:1363-73.

All sleep tests from both HR and VSU were manually scored by specialized technicians. The sleep staging was performed using the standardized AASM criteria apnea was defined as a decrease in the peak signal excursion of ≈ 90% in the airflow signal from the pre event baseline for at least 10 seconds. Hypopnea was defined as a discernible reduction in the amplitude of the airflow signal from the pre event baseline of at least 10 seconds of duration, associated with a 3% fall in SpO2 or an arousal (if PSG).

Regarding CPAP titration, it was performed in-hospital by PSG or by RP with AutoCPAP (Airsense 10, ResMed) with technician manual adjustment when following the HR. Optimal fixed CPAP values were obtained following the AASM recommendations: CPAP pressure was increased progressively until all respiratory events, snore and flow limitation disappeared in all body positions and all sleep stages. Once the physician had determined optimal pressure, patients were provided with a fixed pressure CPAP (Dreamstation, Respironics) to use at home.

In the VSU CPAP titration was remotely performed with Dreamstation, (Respironics). This device is able to show professionals different signals related to the treatment (pressure, leaks, residual AHI, hours of use) through a website (EncoreAnywhere, Respironics). CPAP pressure was determined by AutoCPAP at the beginning of the treatment and maintained ±3 cmH2O. According with these signals, a physiotherapist could remotely adjust CPAP pressure during titration (and also during follow-up if needed).
