# Supplementary material for: Comprehensive management of obstructive sleep apnea by telemedicine: Clinical improvement and cost-effectiveness of a Virtual Sleep Unit. A randomized controlled trial
Source: PLoS One. 2019 Oct 24;14(10):e0224069. doi: 10.1371/journal.pone.0224069 (PMC6812794; doi:10.1371/journal.pone.0224069)
Supplement: S2 Text — (DOCX) [file pone.0224069.s002.docx]

Costs analysis

The costs analysis was performed following the procedure of Isetta V et al Thorax. 2015; 70:1054-61. It included total costs and OSA-related costs. Costs were divided in direct medical and non-medical costs, and indirect costs. Direct medical costs consisted in the use of healthcare resources, staff salaries and pharmaceutical consumption. Direct non-medical costs referred to travel expenses incurred by patients. Indirect costs were those related to the opportunity costs of lost productivity due to medical visits, which were estimated using the human capital approach, and for this, wages were approximated based on the average salary in Spain adjusted by gender and educational level (Spanish Wage Structure Survey, 2015 http://www.ine.es). Data related to costs were collected for the entire process. The costs of the initial visit mainly included the diagnostic sleep studies. Their costs were provided by the administrative department of the Hospital Clinic of Barcelona and included personnel salaries (physicians, nurses, technicians and secretaries), as well as material and maintenance costs: 178.47€ PSG, 102.39€ in-hospital RP, 76.50€ HRP and 89.62€ 3N-HRP. Initial visit also included the costs of any repeated tests and CPAP titration. Follow-up visits included costs of mask interfaces (158.11€ per unit), humidifiers (220€) and adapters (37.62€), pharmaceutical expenses and any material replacement. Travel expenses, opportunity costs of lost productivity incurred by patients, and the time spent attending hospital or the video-conferences were recorded for all visits.

The number of extra visits (general practitioners, specialists, hospital admissions, emergency and intensive care unit visits) were also recorded. Unit costs were provided by the administrative department of the hospital.
