# Supplementary material for: Comprehensive management of obstructive sleep apnea by telemedicine: Clinical improvement and cost-effectiveness of a Virtual Sleep Unit. A randomized controlled trial
Source: PLoS One. 2019 Oct 24;14(10):e0224069. doi: 10.1371/journal.pone.0224069 (PMC6812794; doi:10.1371/journal.pone.0224069)
Supplement: S3 Text — (DOCX) [file pone.0224069.s003.docx]

Cost-effectiveness analysis

The Bayesian cost-effectiveness analysis was carried as described by O’Hagan et al. (Statistics in Medicine, 2001; 20;5: 661-824) and Isetta V et al. (Thorax 2015; 70:1054-61). Considering the asymmetry in the cost distribution and the correlation between effectiveness and cost, a multivariate normal distribution for both effectiveness and log-transformed total and OSA-related costs was assumed. Non-informative proper priors were considered, in particular a multivariate normal prior distribution for the vector of means, with normal marginal with mean zero and an extremely large variance (10^5^), and zero correlation. A Wishart distribution with 4 degrees of freedom and an Indentity scale matrix were considered as the prior distribution for the variance-covariance matrix. Posterior distribution was estimated using Markov Chain Monte Carlo methods. The expected mean effectiveness and costs and 95% Bayesian credible interval were estimated from the posterior distributions. Results were represented by a cost-effectiveness plane, in which the joint posterior distribution of the incremental effectiveness and costs are displayed in an x-y plot, as well as a cost-effectiveness acceptability curve (CEAC), in which the probability of preference for the VSU is displayed as a function of the willingness to pay for a QALY (Vallejo-Torres et al Health Econ. 2018; 27:746-761). Cost-effectiveness analysis was carried out using OpenBUGS software.
