# Supplementary material for: Comprehensive management of obstructive sleep apnea by telemedicine: Clinical improvement and cost-effectiveness of a Virtual Sleep Unit. A randomized controlled trial
Source: PLoS One. 2019 Oct 24;14(10):e0224069. doi: 10.1371/journal.pone.0224069 (PMC6812794; doi:10.1371/journal.pone.0224069)
Supplement: S1 Table — ITT Population. Data are expressed by mean ± SD. AHI: Apnea hipopnea index. CT90%: percentage of time with oxygen saturation <90%. ESS: Epworth sleepiness scale. ODI3%: Oxygen desaturation index 3%. QSQ: Quebec Sleep Questionnaire. QoL: quality of life. VAS: visual analogue scale. *See domains of QSQ for non CPAP and CPAP patients in S2 and S3 Tables. (DOCX) [file pone.0224069.s004.docx]

S1 Table shows the sleep parameters and the results of baseline quality of life and sleepiness questionnaires when considering two subpopulations according whether the patients received or not CPAP treatment. Baseline scores of quality of life and sleepiness questionnaires showed statistically significant baseline differences in EQ-5D and VAS in non-CPAP patients. No differences were found in baseline questionnaires among patients who received CPAP treatment.

**Table S1. Sleep baseline characteristics and questionnaires in non-CPAP and CPAP patients. ITT Population**

|  | **All**  **(n=186)** | **Virtual Sleep Unit**  **(n=94)** | **Hospital routine**  **(n=92)** | **p value** |
| --- | --- | --- | --- | --- |
| **Non-CPAP patients** | **n=114** | **n=62** | **n=52** |  |
| AHI | 14.46 ± 10.61 | 13.20 ± 7.74 | 15.94 ± 13.17 | 0.555 |
| ODI3% | 14.37 ± 11.19 | 13.00 ± 7.08 | 15.92 ± 14.44 | 0.644 |
| CT90% | 6.47 ± 12.38 | 8.49 ± 14.59 | 4.14 ± 8.74 | **0.012** |
| QSQ* | 26.28 ± 4.82 | 25.85 ± 5.19 | 26.78 ± 4.36 | 0.481 |
| EuroQol-5D | 0.83 ± 0.18 | 0.80 ± 0.20 | 0.86 ± 0.16 | 0.132 |
| EuroQol-VAS | 72.62 ± 16.05 | 70.31 ± 15.83 | 75.88 ± 15.77 | 0.485 |
| ESS | 9.25 ± 4.38 | 10.25 ± 4.43 | 8.08 ± 4.06 | **0.010** |
| **CPAP patients** | **n=72** | **n=32** | **n=40** |  |
| AHI | 52.36 ± 25.27 | 47.07 ± 20.53 | 56.56 ± 28.03 | 0.161 |
| ODI3% | 50.93 ± 22.55 | 46.30 ± 20.18 | 54.49 ± 23.87 | 0.114 |
| CT90% | 26.97 ± 20.68 | 29.13 ± 20.51 | 25.26± 20.91 | 0.295 |
| QSQ* | 25.04 ± 5.17 | 25.79 ± 5.47 | 24.44 ± 4.89 | 0.257 |
| EuroQol-5D | 0.79 ± 0.19 | 0.79 ± 0.17 | 0.79 ± 0.21 | 0.995 |
| EuroQol-VAS | 67.79 ± 20.31 | 70.97 ± 18.05 | 65.27 ± 21.84 | 0.253 |
| ESS | 11.21 ± 5.08 | 9.69 ± 4.22 | 12.43 ± 5.42 | 0.080 |

Data are expressed by mean ± SD. AHI: Apnea hipopnea index. CT90%: percentage of time with oxygen saturation <90%. ESS: Epworth sleepiness scale. ODI3%: Oxygen desaturation index 3%. QSQ: Quebec Sleep Questionnaire. QoL: quality of life. VAS: visual analogue scale.

*See domains of QSQ for non CPAP and CPAP patients in Tables S2 and S3.
