# Supplementary material for: Comprehensive management of obstructive sleep apnea by telemedicine: Clinical improvement and cost-effectiveness of a Virtual Sleep Unit. A randomized controlled trial
Source: PLoS One. 2019 Oct 24;14(10):e0224069. doi: 10.1371/journal.pone.0224069 (PMC6812794; doi:10.1371/journal.pone.0224069)
Supplement: S2 Table — Comparison of Virtual Sleep Unit and Hospital routine. Patients without CPAP indication after sleep study from per protocol population. Data are expressed by mean ± SD. LS mean analysis is based on an ANCOVA model adjusted by age, sex and AHI for change from baseline to follow-up in the questionnaire variables as response to treatment group in per protocol population.ANCOVA: analysis of covariance. BMI: body mass index. CI: confidence interval. ESS: Epworth sleepiness scale. LS mean: least square mean. QSQ: Quebec Sleep Questionnaire. QoL: quality of life. VAS: visual analogue scale. (DOCX) [file pone.0224069.s005.docx]

It was observed that in non-CPAP patients (S2 Table) overall QSQ was similar in both randomization groups, although those who followed the HR presented a significant slightly improvement in its social interactions domain (-0.461; 95%CI; -0.877 to -0.045; p=0.030). By contrast, non-CPAP patients in the VSU presented an improvement in EQ-VAS (5.583; 95%CI; 0.343 to 10.823; p=0.037). Mean improvement of ESS after follow-up was similar in both groups after follow-up.

**Table S2. Quality of life and sleepiness questionnaires of non-CPAP patients. Comparison of Virtual Sleep Unit / Hospital routine**

|  | **Virtual Sleep Unit**  **(n=53)** | | **Hospital routine**  **(n=46)** | | **LS mean difference (Virtual Sleep Unitminus Hospital routine)** | **95% CI for the difference** | | |
| --- | --- | --- | --- | --- | --- | --- | --- | --- |
|  | **Baseline** | **Follow-up** | **Baseline** | **Follow-up** |  | **Lower limit** | **Upper limit** | **p value** |
| Total QSQ | 25.70 ± 5.42 | 26.22 ± 5.56 | 27.01 ± 4.38 | 28.22 ± 4.34 | -0.801 | -2.021 | 0.419 | 0.196 |
| Daytime hypersomnia | 5.26 ± 1.38 | 5.44 ± 1.27 | 5.81 ± 1.14 | 6.00 ± 0.97 | -0.034 | -0.369 | 0.300 | 0.839 |
| Diurnal symptoms | 4.77 ± 1.44 | 4.93 ± 1.41 | 5.26 ± 1.46 | 5.49 ± 1.25 | -0.116 | -0.454 | 0.222 | 0.498 |
| Nocturnal symptoms | 4.81 ± 1.15 | 5.13 ± 1.20 | 5.06 ± 1.16 | 5.34 ± 1.07 | 0.036 | -0.328 | 0.400 | 0.845 |
| Emotions | 5.41 ± 1.22 | 5.43 ± 1.20 | 5.61 ± 0.98 | 5.83 ± 0.98 | -0.225 | -0.511 | 0.060 | 0.120 |
| Social interactions | 5.45 ± 1.42 | 5.29 ± 1.28 | 5.27 ± 1.18 | 5.56 ± 1.11 | -0.461 | -0.877 | -0.045 | **0.030** |
| EuroQol-5D | 0.80 ± 0.19 | 0.85 ± 0.17 | 0.87 ± 0.15 | 0.86 ± 0.16 | 0.048 | 0.007 | 0.104 | 0.085 |
| EuroQol-VAS | 70.23 ± 16.55 | 75.11 ± 14.12 | 77.72 ± 14.33 | 77.67 ± 14.60 | 5.583 | 0.343 | 10.823 | **0.037** |
| ESS | 10.49 ± 4.29 | 9.15 ± 4.85 | 8.11 ± 4.16 | 7.52 ± 4.63 | -0.658 | -1.877 | 0.561 | 0.286 |

Patients without CPAP indication after sleep study from per protocol population. Data are expressed by mean ± SD. LS mean analysis is based on an ANCOVA model adjusted by age, sex and AHI for change from baseline to follow-up in the questionnaire variables as response to treatment group in per protocol. population.ANCOVA: analysis of covariance. BMI: body mass index. CI: confidence interval. ESS: Epworth sleepiness scale. LS mean: least square mean. QSQ: Quebec Sleep Questionnaire. QoL: quality of life. VAS: visual analogue scale.
