# Supplementary material for: Comprehensive management of obstructive sleep apnea by telemedicine: Clinical improvement and cost-effectiveness of a Virtual Sleep Unit. A randomized controlled trial
Source: PLoS One. 2019 Oct 24;14(10):e0224069. doi: 10.1371/journal.pone.0224069 (PMC6812794; doi:10.1371/journal.pone.0224069)
Supplement: S3 Table — Comparison of Virtual Sleep Unit and Hospital routine. Patients under CPAP treatment after sleep study from per protocol population. Data are expressed by mean ± SD. LS mean analysis is based on an ANCOVA model adjusted by age, sex and AHI for change from baseline to follow-up in the questionnaire variables as response to treatment group in per protocol population. ANCOVA: analysis of covariance. BMI: body mass index. CI: confidence interval. ESS: Epworth sleepiness scale. LS mean: least square mean. QSQ: Quebec Sleep Questionnaire. QoL: quality of life. VAS: visual analogue scale. (DOCX) [file pone.0224069.s006.docx]

Patients under CPAP treatment managed in the hospital presented a greater improvement in total QSQ score (-3.017; 95%CI; -5.230 to -0.804: p=0.009) as well and in its daytime hypersomnia and social interactions domains (-0.827; 95%CI; -1.519 to -0.135; p=0.020 and -0.758; 95%CI; -1.410 to -0.105; p=0.024, respectively). There were no significant differences in quality of life improvement measured by EQ-5D or EQ-VAS. Similarly to QSQ, ESS improvement was significantly higher in patients under the HR (3.954; 95%CI; 1.517 to 6.390; p=0.002).

**Table S3. Quality of life and sleepiness questionnaires of patients under CPAP treatment. Comparison of Virtual Sleep Unit and Hospital routine**

|  | **Virtual Sleep Unit**  **(n=27)** | | **Hospital routine**  **(n=28)** | | **LS mean difference (Virtual Sleep Unitminus Hospital routine)** | **95% CI for the difference** | | |
| --- | --- | --- | --- | --- | --- | --- | --- | --- |
|  | **Baseline** | **Follow-up** | **Baseline** | **Follow-up** |  | **Lower limit** | **Upper limit** | **p value** |
| Total QSQ | 25.9 ± 5.27 | 29.35 ± 4.27 | 24.63 ± 0.73 | 30.85 ± 3.16 | -3.017 | -5.230 | -0.804 | **0.009** |
| Daytime hypersomnia | 5.43 ± 1.15 | 6.01 ± 0.92 | 5.30 ± 1.26 | 6.57 ± 0.44 | -0.827 | -1.519 | -0.135 | **0.020** |
| Diurnal symptoms | 5.15 ± 1.28 | 5.65 ± 1.15 | 5.03 ± 1.56 | 6.02 ± 0.93 | -0.537 | -1.210 | 0.136 | 0.115 |
| Nocturnal symptoms | 4.51 ± 1.34 | 5.84 ± 1.00 | 4.19 ± 1.25 | 6.05 ± 0.81 | -0.531 | -1.219 | 0.156 | 0.127 |
| Emotions | 5.49 ± 1.09 | 5.90 ± 0.90 | 5.33 ± 1.11 | 6.06 ± 1.10 | -0.364 | -0.782 | 0.055 | 0.208 |
| Social interactions | 5.32 ± 1.29 | 5.95 ± 1.00 | 4.78 ± 1.33 | 6.15 ± 1.07 | -0.758 | -1.410 | -0.105 | **0.024** |
| EuroQol-5D | 0.79 ± 0.17 | 0.84 ± 0.20 | 0.79 ± 0.21 | 0.84 ± 0.17 | 0.021 | -0.067 | 0.108 | 0.637 |
| EuroQol-VAS | 70.93 ± 17.71 | 76.74 ± 12.96 | 66.85 ± 20.25 | 70.44 ± 20.97 | 3.123 | -8.950 | 15.196 | 0.605 |
| ESS | 10.11 ± 3.66 | 7.22 ± 3.21 | 12.43 ± 5.81 | 6.29 ± 3.69 | 3.954 | 1.517 | 6.390 | **0.002** |

Patients under CPAP treatment after sleep study from per protocol population. Data are expressed by mean ± SD. LS mean analysis is based on an ANCOVA model adjusted by age, sex and AHI for change from baseline to follow-up in the questionnaire variables as response to treatment group in per protocol population. ANCOVA: analysis of covariance. BMI: body mass index. CI: confidence interval. ESS: Epworth sleepiness scale. LS mean: least square mean. QSQ: Quebec Sleep Questionnaire. QoL: quality of life. VAS: visual analogue scale.
