# Supplementary material for: Comprehensive management of obstructive sleep apnea by telemedicine: Clinical improvement and cost-effectiveness of a Virtual Sleep Unit. A randomized controlled trial
Source: PLoS One. 2019 Oct 24;14(10):e0224069. doi: 10.1371/journal.pone.0224069 (PMC6812794; doi:10.1371/journal.pone.0224069)
Supplement: S4 Table — (DOCX) [file pone.0224069.s007.docx]

There were no differences between the number of total recorded extra visits and the number of material replacements for the 6 months of follow-up in both treatment groups (S4 Table).

**S4 Table. Total number of extra visits and total number of CPAP material replacements**

|  | **Virtual Sleep Unit** | **Hospital routine** | **p value** |
| --- | --- | --- | --- |
| **Extra visits** | | | |
| General practitioner visits | 37 | 47 | 0.744 |
| General practitioner visits (OSA-related) | 4 | 6 | 0.698 |
| Specialist visits | 49 | 41 | 0.439 |
| Specialist visits (OSA-related) | 11 | 9 | 0.642 |
| Hospital admissions | 5 | 4 | 0.503 |
| Emergency department visits | 4 | 12 | 0.460 |
| Intensive care visits | 0 | 1 | 0.312 |
| **Material replacements** | | | |
| Mask interfaces | 3 | 4 | 0.679 |
| Humidifiers | 14 | 17 | 0.283 |
| Adapters | 1 | 4 | 0.166 |
