# Supplementary material for: Comprehensive management of obstructive sleep apnea by telemedicine: Clinical improvement and cost-effectiveness of a Virtual Sleep Unit. A randomized controlled trial
Source: PLoS One. 2019 Oct 24;14(10):e0224069. doi: 10.1371/journal.pone.0224069 (PMC6812794; doi:10.1371/journal.pone.0224069)
Supplement: S5 Table — (DOCX) [file pone.0224069.s008.docx]

**S5 Table: Degree of satisfaction**

|  | **Virtual Sleep Unit** | **Hospital routine** |
| --- | --- | --- |
| **Degree of satifaction** | | |
| Indifferent | 1,3% | 1,4% |
| Good | 41,8% | 32,9% |
| Very good | 57% | 65,8% |
| **Access to professionals** | | |
| Easy | 41,8% | 31,5% |
| Very easy | 58,2% | 68,5% |
| **Understanding the instructions** | | |
| Regular | 1,3% | 0% |
| Well | 26,6% | 13,7% |
| Very well | 72,2% | 86,3% |
| **VSU Patient satisfaction and evaluation of the procedure** | | |
| **Repeat the procedure** | |  |
| Low/moderate | 10% |  |
| High/very high | 90% |  |
| **Recommend to other people** |  |  |
| Low/moderate | 5% |  |
| High/very high | 95% |  |
| **Comfortable during the interview** | |  |
| Low | 1,2% |  |
| Moderate | 20% |  |
| High/Very high | 78,8% |  |
| **Preference at hospital** | |  |
| None | 67,5% |  |
| Some times | 30 % |  |
| Yes | 2,5% |  |
| **Adequate intimacy during the interview** | |  |
| Low/moderate | 6,3% |  |
| High/very high | 93,7% |  |
