# Supplementary material for: Comprehensive management of obstructive sleep apnea by telemedicine: Clinical improvement and cost-effectiveness of a Virtual Sleep Unit. A randomized controlled trial
Source: PLoS One. 2019 Oct 24;14(10):e0224069. doi: 10.1371/journal.pone.0224069 (PMC6812794; doi:10.1371/journal.pone.0224069)

2014

**Applicability, efficacy and cost effectiveness of the sleep apnea hypopnea syndrome (SAHS) management by using the technology based on information and communication (TIC's)**

**SUMMARY**

This project develops a new form of management of subjects with suspected SAHS and different sleep disorders by applying TIC's. It is the response to the increased detection of patients with SAHS. If there is more cases of suspected SAHS will be more typical cases with problems as well as complex patients that require sleep specialist. Due to the health cost at present these patients must be managed in a cost-effective way. TIC's could be a response. Objectives. 1) Analysis of efficacy and cost effectiveness of two management programs of SAHS. The classic hospital one versus another based on work using ICT. The main variable is the Quebec questionnaire and in cases of CPAP the compliance as the analysis of EuroQol-5D, Epworth, the number side effects, and satisfaction in both groups. Design: Prospective, randomized, controlled, open, parallel in 202 patients (101 per arm). Monitoring will be 6 months and 4 assessments. Statistical analysis: Effectiveness: Changes in the Quebec scale is compared before and after intervention between the two arms using analysis of ANCOVA (premise 2-point improvement in the Telematic group). The other variables, compliance as the analysis of other EuroQol-5D, Epworth were analyzed using independent differences in means (or nonparametric equivalent) or Chi2 for dichotomous variables. Cost-effectiveness: the costs generated by either program will be measured against the effectiveness of the main variables by calculating the ICER following Bayesian techniques

**INTRODUCTION**

This project is aimed at developing a new form of management of subjects suspected of suffering from sleep apneas-hypopneas syndrome (SAHS) and sleep disorders in general. In clinical practice these patients can be divided into 3 large groups: A) Those with a high "pre-test" (with typical signs/symptoms without co-morbidities); B) Patients with suspected SAHS but with significant associated pathology (fibromyalgia, insomnia or other sleep pathology) that may hinder their assessment and require special techniques; and C) Subjects with low pre-test, or special populations (advanced age, pregnancy, stroke, severe heart disease etc.) that are not always easy to guide.

These last two groups frequently consult at different levels of care requiring many tests and their diagnosis is always made in third level units. Group A is easier to care for and it is undoubtedly essential that its management takes place outside the hospitals or at least those of non reference. However, this is not the case for groups B and C. Given that SAHS has penetrated society and among health professionals, there are now a greater number of patients diagnosed from all the groups mentioned. This entails or will entail a series of problems, for example, in the treatment with CPAP of typical patients or high pre-test. In this case, although the proportion of subjects with problems during CPAP treatment (low compliance, local problems, persistence of drowsiness, etc.) will be the same, the total number of patients will be greater.

In addition, as already mentioned, SAHS will coexist with several associated diseases such as severe cardiac pathology or fibromyalgia, or in different populations such as the elderly, pregnant women, children, etc. All these aspects make many patients difficult to manage from a clinical point of view and, consequently, require professionals more specifically prepared to attend to them. With the idea that when a disease is frequent all levels of care must be involved, this project aims to develop a new way of caring for and managing these patients with a high level of quality, adequate compliance and that the procedure is cost-effective. To do this, it is proposed to work through the application of TIC´s, with virtual visits and home studies basically with telemedicine techniques as a thread of care.

This project is the result of the group's own trajectory to diagnose patients with SAHS that began in 1997 with the description of the usefulness of simplified systems. (Parra et al. ERJ 1997, Lloberes et al AJRCCM 1996). Then, using this system for several years in three multicenter studies published by the Spanish sleep group, the previous proposals have been consolidated (Masa et al AJRCCM 2011, Thorax 2011, AJRCCM 2004). We have recently established the usefulness of telemedicine in pilot studies (Isetta et al. EJMR 2013). In this way, and as a result of all previous experience, we believe we are in a position to propose a project aimed at a real transfer in daily practice.

SAHS is very prevalent. It is defined as a picture of symptoms that alter the quality of life together with metabolic, neurological and cardiovascular disorders (1-6) secondary to an abnormal number of episodes of upper airway obstruction. It is associated with traffic accidents (7) and is associated with excess mortality (8). Under-diagnosed patients consume more health resources (9) and it is considered a public health problem of the first magnitude (1). The increase in their knowledge has led to an increase in the demand for consultations and the need for more diagnostic studies; however, in many cases they have not been accompanied by new strategies for diagnosis, treatment and monitoring of the disease. Furthermore, the fact that SAHS is frequently associated with other pathologies result that it is considered a chronic disease (10) that makes it necessary to adopt a new approach to its management in order to make it more viable, cost-effective and, above all, effective. Two points are raised as general concepts to consider:

1. Care levels. As with COPD, and given its prevalence, in SAHS all the health levels must be involved, from family medicine, nurses, general specialists, professionals with expertise in sleep disorders to companies providing respiratory therapies. In other words, networking. Our group has experience in this area, as we started this type of study as early as 2007 we started this type of studies. We showed how general pneumologists (with rotation in sleep during residence) working in non-reference hospitals or in primary centers in connection with the hospital sleep unit, adequately handle many patients with SAHS and especially requesting the corresponding tests in an appropriate way (conventional polysomnography or respiratory - home and hospital - and oximetry) (11,12). This is undoubtedly a very important part of the management of patients with high pre-test.
2. Telemedicine or the application of new information and communication technologies (ICTs) to clinical care. In recent years, medical technology has been widely applied and has been boosted by the incorporation of effective innovations arising from various areas such as bioengineering. One of the few exceptions to this rapid process of application or adaptation to technological advances is found in the medicine of everyday life. Its implementation and penetration in clinical practice is much lower than in other scientific-professional fields or even in our daily lives (13). The health care deficit through telemedicine systems is surprising, especially in sleep pathology, especially in the field of SAHS where new protocols are urgently needed to alleviate its serious health care crisis: under-diagnosis, comorbidity and long waiting lists. Some initiatives have been develop although few, both in the US and in Europe and also in Spain, (14) although very timidly. This project aims to routinely apply TIC´s in the clinical practice of patients with suspected SAHS. A very significant point is the fact that it is necessary to adapt telemedicine to the type of patients, since its application to certain populations may not be effective, quite the opposite. Recently, increased morbidity or even mortality has been described in older multimorbid patients or patients with COPD (Takahashi et al. Arch Intern Medicine 2012, Hallet et al. Ann of Internal Medicine 2012). From our experience in recent pilot studies, patients with suspected SAHS may benefit greatly from ICTs. In order to fully manage the diagnosis, treatment and follow-up procedure in an innovative way (Figure 1), we have carried out a series of pilot studies ranging from training to treatment follow-up, these experiences being very positive, allowing us to face a global process (15).

Currently the majority of patients are followed in the reference centers (Figure 2-A) and obviously in the current context is not logical. Non-complex patients will be managed outside the hospital (Figure 2-B). However, complex cases, such as poor compliance, morbid obesity, surgical treatments, Cheyne Stokes, servoventilation, advanced age or severe associated pathologies, among others, should be treated in sleep units, and it is to be expected that in the future their number may increase significantly (Figure 2-C) and hence that hospital professionals should adapt to this changing reality and develop systems to care for this type of patients, ideally outside the hospital setting to optimize cost effectiveness. ICTs are an option since they allow the use of: a) appropriate diagnostic techniques (out-of-hospital PSG), b) studies of several days as we have recently published -type Holter-) (16) and c) teleconsultations (Figure 1). All of this is managed by sleep specialists (16) as we have verified that it is a very important option as it facilitates consultations that require less time, avoids trips for patients (loss of working hours or their distant residence or their condition prevents them from moving easily). In addition, occasional visits will improve compliance (15).

As a summary, it can be said that in order to settle positively in this difficult and changing situation such as the current one, different strategies of action must be developed. Networking is an option, telemedicine techniques are also an option and the combination of both will probably be the optimal way to arrive at a personalized and cost-effective medicine in which all the care links intervene. Our experience allows us to undertake this broad project that aims to manage the entire process of care to the SAHS in a different way and that fits the reality in part related to what is known as the 4 P's of medicine: 1. Personalized (depends on genome characters) 2. Predictive from individual information that can establish predictions 3. Preventive, from predictions implement preventive measures and 4. Participatory by the patient. For the development of this project, the efficiency and cost-effectiveness will be compared between a traditional system and another that basically uses ICTs (Table 1). In order to do so, two options could be chosen: 1) a study only on the "difficult" population (but in this case the behaviour of the high pre-test group would not be known, which could be very different), or 2) which is the one we have chosen, to study all types of patients, without assessing their initial characteristics. We believe that the latter is better because it will be more real and, above all, that in the subsequent analysis the various groups mentioned will already be analyzed.

**REFERENCES**

1.Durán-Cantolla et al. Documento de consenso nacional sobre el SAHS. Arch Bronconumol 2005; 41:1-110.

2.Durán J et al. Obstructive sleep apnea-hypopnea and related clinical features in a population-based sample of subjects aged 30 to 70 years. Am J Respir Crit Care Med 2001; 163:685-9.

3.Baldwing et al. The association of sep-disordered breathing and sleep symptoms with quality of life in the Sleep Heart Healt Study. Sleep 2001; 24:96-105.

4.Martínez-García MA et al. Effect of CPAP on blood pressure in patients with obstructive sleep apnea and resistant hypertension: the HIPARCO trial. JAMA. 2013; 11; 310:2407-15.

5.Somers VK et al. Sleep apnea and cardiovascular disease. Circulation. 2008; 118:1080-111.

6.Yaggi HK et al. SAHS a a risk factor for stroke and death. NEJM 2005; 353:2034-41.

7.Terán-Santos J et al. The association between SAHS and the risk of traffic accidents. NEJM 1999; 340:847 -51.

8.Young T et al. Sleep-disordered-Breathing and mortality; Eigtheen-year follow-up of Wisconsin Sleep Cohort. Sleep 2008;31:1071-8.

9.Diaz K, et al. Obstructive sleep apnea is associated with higher healthcare utilization in elderly patients. Ann Thorac Med. 2014; 9:92-8.

10.Heatley EM et al Obstructive sleep apnoea in adults: a common chronic condition in need of a comprehensive chronic condition management approach. Sleep Med Rev. 2013; 17:349

11.Hernández L, et al.Management of sleep apnea: concordance between nonreference and reference centers. Chest. 2007;132:1853-7.

12.Chamorro N et al. An integrated model involving sleep units and primary care for the diagnosis of sleep apnoea Eur Respir J. 2013; 42:1151-4

13.Ramon Farre. Telemedicina y enfermedades respiratorias durante el sueño: perspectivas de futuro. ArchBronconeumol.2009;45(3):105 -106

14.Coma-Del-Corral MJ et al. Reliability of telemedicine in the diagnosis and treatment of sleep apnea syndrome. Telemed J E Health. 2013; 19:7-12

15.Isetta V et al. Telemedicine-based approach for obstructive sleep apnea management: building Evidence. Interact J Med Res. 2014; 19;3(1):e6. doi: 10.2196/ijmr.3060.

16.Guerrero A, et al. Management of Sleep Apnea without high pre-test probability or with comorbidities by three nights of portable sleep monitoring. Sleep August 2014

17.Negrín MA et al. A fully Bayesian cost-effectiveness analysis using conditionally specified prior distributions. In Health Care Costs: Causes, Effects and Control, 2008. pp. 1-17 (Chapter 7). Martel.

**HYPOTHESIS**

1. The efficiency of the management of the SAHS using a work based on TIC's will be optimal to the one currently used in the hospital framework that uses both respiratory polygraph or PSG, including all types of patients from high probability to the most complex.
2. -The cost-effectiveness of the management of SAHS using a work based on ICT's is optimal than that currently used in the hospital framework that uses both respiratory polygraph or PSG, including all types of patients from high probability to the most complex.
3. -Optimal cost-effectiveness will occur especially in complex patients. These patients will directly contact the reference centre which, through the use of ICT, will simplify the process. These same groups of patients when they follow the usual process require several visits at different levels of care and always end up being diagnosed in the referral centers.
4. -The transfer of the telematic process will be possible and can be quickly included in the care routine.

**OBJECTIVES**

1. -Evaluation of two SAHS management programs, the classic one currently used as opposed to another based on work using ICT's taking the Quebec questionnaire as the main variable and in cases with CPAP the compliance of the treatment.

2. -Analysis of the cost-effectiveness of the programs mentioned above, valuing the Quebec questionnaire as the main variable and, in the cases of CPAP, compliance with treatment.

-Effectiveness of the program based fundamentally on technology between the group of complex patients (Groups 2 and 3 initially described) and the high possibility of suffering from an SAHS (Group 1). Questionnaires: Epworth and EuroQol-5D. Number of complications or side effects. 3. Patient satisfaction questionnaires

**DESIGN**

DESIGN AND SUBJECTS OF THE STUDY (see figures at the end).

A randomized, open-ended, parallel patient group study in which an analysis of treatment efficacy and cost-effectiveness will be performed. All patients with suspected SAHS diagnosis who belong to the primary care centers (CAP) of the Esquerra del Eixample area of Barcelona, which corresponds to the health area of the Hospital Clínic of Barcelona, will be included consecutively. Patients with: a) psycho-physical incapacity to carry out questionnaires; b) difficulty in using communications technology (smartphone), c) pregnancy, d) failure to obtain informed consent, e) severe or disabling drowsiness or f) unstable illnesses will be excluded. Patients will be randomized, following a sequence generated by a database, into two groups (Table 1):

- Group 1: Patients who follow the usual procedure (hospital). After the visit in the primary physician/specialist out-of-hospital who will make a decisions. Can refer or not the patients to the hospital for a sleep test, titration if appropriate and follow-up for 6-12 months in the hospital (SEPAR regulations).
- Group 2: Telemedicine monitoring by the referral centre: this group of patients will be detected in the outpatient clinic in the same way as group 1 but will carry out the diagnosis, education, treatment and monitoring by means of a totally out-of-hospital telematic procedure carried out by sleep doctors. To minimize possible information biases derived from the open procedure, the professional in charge of introducing and evaluating the questionnaires as well as other main objective variables (measurement of CPAP compliance) will not know the patient's group of belonging.

**WORKING SCHEME:**

**Variables**

The following data shall be collected on patients who have passed the inclusion/exclusion criteria:

1) Anthropometric and clinical data related to SAHS. Symptoms related to other sleep disorders. Physical examination specifically maxillofacial ENT. Blood pressure. 2) Compliance with treatment if CPAP is used (meter reading and optimal pressure time).

Given the characteristics of the project and with the aim of simplifying the protocol which, due to its characteristics, must be simple, only a few additional variables will be collected, specifically:

- Quebec questionnaire (Lacasse and Thorax 2004), Spanish translationMedicina Clinica Merrejon 2012).
- EuroQol-5D (Eurocold group; health Policy 1990).
- Epworth sleepiness scale (Johns MW Sleep 1991) and
- Satisfaction scale and side effects. (Self-administered with 15 minutes of time for completion)

**Proceedings:**

Sleep studies and CPAP titration. 1) PSG or hospital respiratory polygraph (PR): The technique and analysis will be carried out according to AASM 2012-SEPAR 2011 regulations. 2) PSG or PR home. The technique and analysis will also conform to the aforementioned standardized regulations. The home care nursing group for chronic patients will also deliver the equipment. In this case the data will be transmitted to the hospital via telematics. For CPAP titration the same procedure will be followed except that the company will provide the automatic equipment for titration, the training will be done from the hospital via telematics and if it is a difficult titration will be adjusted for several days according to results observed telematrically. In the usual treatment arm the professionals of the traditional centres will follow the already established processes. Valid studies for PSG or PR will be considered those that comply with the regulations of the Spanish sleep group. As far as treatment is concerned, the SEPAR regulations will be followed (Lloberes et al. 2011).

Follow-up: Patients will be evaluated according to SEPAR general regulations (onset, at 1, 3 months, and 6 months) and above all depending on the medical criteria. Obviously the extra visits made will be valued. The aforementioned questionnaires will be collected and, in the case of CPAP, compliance at the beginning and at the end (CPAP compliance is not collected in the intermediate months because a group is telematic).

**Justification of sample size.** The main study variable is the change in the score of the dimension of the Quebec questionnaire that requires a higher "n". It is estimated that for an alpha error of 0.05, beta of 0.20 and a clinically relevant difference (change in total score ≥2 points on the questionnaire) between the two study groups, a minimum of 101 patients per randomization arm (202 patients in total) is required. An estimated 20% loss (between those who lose and those who do not tolerate CPAP), according to published data on the degree of compliance with CPAP in Spain is estimated at 75-80%.

**Statistics:**

Main objectives: After a filtering of the data collected, the variables of the study will be tabulated in a database. A specific unique code will be assigned to identify the patients. The connection key between the code and the subject will be in the hands of the IP who will assume all responsibility for data protection and care. The construction of the database, evaluation of questionnaires, entry of data into the database and analysis of the results will be carried out by personnel external to the research. The construction of the database and analysis of the data will be carried out by computer professionals and statisticians respectively. Univariate study: In the case of qualitative variables, their frequency and valid percentage will be determined. The lost values will be taken into account in order to give the results. For quantitative variables, measures of central tendency, position and dispersion will be included.

**Effectiveness assessment.**

Statistical analysis shall be based on the intention-to-treat principle. The comparison in the changes in the score of the QUEBEC questionnaire (6 months vs. Basal) between both groups will be carried out from the ANCOVA test, obtaining the mean scores at 6 months for each group adjusted by the basal values and other confusing variables such as the BMI, age or the severity of the SAHS. This test will be performed under the assumption of "least squares". Sub-analysis will be performed with the most complex patients in which the same analysis will be replicated. An analysis per protocol will also be carried out in the case of patients with CPAP depending on whether the compliance is less than or not at 4 h/night. For patients without the last follow-up visit, and therefore only with baseline information, the same baseline value will be imputed to the 24-week visit as it is the most conservative option and against the hypothesis of the potential benefit of CPAP intervention. A significant difference of <0.05 will be established for all tests (SPSS version 20 will be used).

**Cost-effectiveness**:

The costs generated by both protocols will be evaluated against the effectiveness of the main variable Quebec, also as secondary (Epworth and EuroQol-5D), using Bayesian cost-effectiveness analysis techniques. Bayesian techniques are especially suitable for this project where the experience of the decision-maker can be incorporated into the study by means of an a priori probability distribution that adequately reflects his or her judgments on the variables studied. In addition, we will avoid the usual assumptions of normal costs and we will be able to use asymmetric costs closer to real practice. This will lead us to the use of Markov Chain Monte Carlo (MCMC) techniques for the simulation of the measures of interest: the cost-effectiveness ratio of each treatment, the incremental cost-effectiveness ratio (ICER), he cost-effectiveness plan, the the net benefit of each treatment, the incremental net benefit (NB) and the cost-effectiveness acceptability curve. Each of these measures has its natural interpretation in terms of the probability and willingness to pay of the decision-maker in the face of an increase in the patient's state of health, making them especially attractive due to their practical applicability. The statistical-mathematical process is not simple, but in this project, we will design a set of codes of easy computer implementation in such a way that the decision-maker obtains in an easy and simple way these measures of interest for later decision making (17).

Secondary objectives: Comparison between both arms of the cited questionnaires (Epworth, EuroQol-5D, Satisfaction and number of side effects). In addition to having other variables of interest collected from each patient, these variables may act as covariates that help minimize uncertainty in the estimation of effectiveness and incremental cost. We therefore propose a series of secondary objectives linked to the proposed Bayesian methodology: 1) to develop a cost-effectiveness for more than one measure of effectiveness, also proposing clear decision-making measures that are easy to interpret and 2) to develop a BMA model (Bayesian Model Averaging) for estimating cost-effectiveness. The BMA solution is obtained as an average of the coefficients obtained by all the possible combinations of the covariates weighted by the a posteriori probability of each model. This implies the selection of which variables have predictive capacity since the choice of a single model among all the possible ones does not consider the uncertainty associated with the choice of the correct model. This objective will allow us to innovate this type of study since we will automatically select the relevant variables by means of the Bayesian selection of covariates (17).

Logistics: a) Protocol: based on the present memory; b) Procedure notebook: explains step by step the development of the study and contains all the questionnaires, tests and how to carry them out for standardization, c) Data collection notebook containing the variables to be collected distributed in the visits. The order and form is the same as that of the database; d) Database with domain belonging to the requesting centre.

Ethical aspects: The study will be carried out in accordance with the guidelines and principles of the Declaration of Helsinki and will be approved by the Ethics and Clinical Research Committee. All study subjects will provide informed consent in writing prior to participation. The quality assurance procedure will be permanent (data management, data security procedures are sufficient, effective and well documented).

Advantages and limitations: The fundamental advantage of this protocol is that it corresponds to reality. Many investigations compare studies in reference centers with only PSG vs. a home system. It is obvious that the cost-effectiveness analysis will always be favourable to the home, as more than 60% of the patients who come to the hospital are resolved with respiratory polygraphs. In addition, once the protocol has been implemented, the intervention of the project manager will be of precise observation without intervening at any time in the different links. On the other hand, the choice of a Bayesian methodology for cost-effectiveness analysis presents us with comparative advantages with classical techniques based on the normal approach: use of asymmetric costs, use of continuous and/or discrete effects, natural interpretability of the measures of interest in terms of probability, etc.

Limitations or problems: a) Volume of patients. Although it is high we do not expect to have problems in the inclusion because among the diverse ambulatories that work in our air, 180 patients are received every year. b) Knowledge of computer science. To be able to participate it is necessary to know how to use basic techniques of internet, computer, smartphobe and/or tablet. We estimate that 25% of the population in our country does not use it. In this sense this point does not invalidate the work at all as the trend is clearly towards reducing this percentage to minimum values. c) Follow-up: to keep "alive" the project has provided for an independent external evaluation every 6 months as well as meetings with the doctors of the ambulatory.

**EXPERIENCE OF THE GROUP.**

The multidisciplinary Sleep Unit of the Hospital Clínic de Barcelona carries out a prolific healthcare activity in which the following services contribute: Pneumology, Neurology, Ear, Nose, Throat, Maxillofacial Surgery, Dietetics, Psychology, Psychiatry and Nursing. Since 1990, as a result of an agreement between the Hospital Clínic and the Institut Català de la Salut (ICS), it has been a Reference Centre for the diagnosis of sleep-related respiratory disorders. In addition, the unit is equipped with all the necessary material to carry out sleep studies (polysomnography, respiratory polygraphs, technicians and secretary). 1500-1700 studies are carried out annually. With regard to research, the unit carries out its activities within the framework of IDIBAPS and the Faculty of Medicine of the University of Barcelona. Its main objective is to improve knowledge of SAHS. The studies carried out are focused on two different aspects: clinical and the most basic or technological. The clinical studies are carried out in collaboration with the Spanish group of respiratory pathology of sleep (the applicant of this project is currently the coordinator).

The group also collaborates with other groups from the University of Barcelona, especially in technological studies and animal models with members of the Biophysics and Bioengineering Unit (Dr. R. Farré and Dr. D. Navajas). As in the clinical aspects, in basic and technological research several studies have been published in journals of great impact (see publications IP curriculum) and highly referenced in the medical literature.

The lines of work have been: 1) Multicentric studies with the Spanish group of sleep pathology, 2) Physiological aspects and consequences of SAHS (Effectiveness of CPAP) 2), Technological aspects (development of a mechanical model to know the functioning of the different automatic CPAP, validation of the nasal probe as an optimal method of measuring flow, 3) In recent years we have already begun the study of mechanisms through biological mediators and models of rat and mouse to deepen and take a further step in the study of the respiratory pathology of sleep through a vision of basic elements of the disease.

In recent years, as a result of a restructuring of the health area, a group of outpatient clinics have been managed jointly with the Hospital Clínic (Esquerra del Eixample Area of Barcelona).

Regarding the cost-effectiveness analysis, which is one of the main outcomes of this project, the group will collaborate with the team of Prof. Francisco Jose Vazquez Polo. The TEBADM research group is attached to the University of Las Palmas de Gran Canaria and has extensive research experience in the field of Bayesian Statistics in general and cost-effectiveness (Health Economics) in particular. The number and quality of its publications guarantee the capacity of the research team to carry out this new project. By way of example and in both research topics, in the last 5 years the group has published some papers in international journals (all of them JCR): ASTIN Bulletin (2012, 2013), Bayesian Analysis (2009, 2010), Communications in Stistics: Theory and Methods (2011), Computational Statistics and Data Analysis (2010), European Journal of Health Economics (2013 accepted), European Journal of Operational Research (2010, 2012, 2013). With regard to national and international groups that work in related areas, the following stand out: (1) Direct contacts with first-level international groups. The group works in the area of Bayesian model selection (groups based in the Italian universities of Padova, Cagliari and Pavia, 2) with respect to recently obtained competitive projects such as: Bayesian Statistical Techniques for Economic Decision Making. Contributions and Applications to the following fields: Actuarial Statistics, Cost-Effectiveness Analysis for Clinical Data and Auditing of Accounts. Ministry of Education and Science (SEJ2006-12685). 2006-09, finally (3) the group also works on new statistical methodologies for economic decision-making in the field of health economics (Consejería de Innovación, Ciencia y Empresa de la Junta de Andalucía mediante técnicas de inteligencia artificial

**TECHNOLOGICAL IMPACT**.

The project proposes the development of a system with innovations in patient management using technology that fits into the field of current transfer research. These advances have potential commercial interest and are candidates for transfer to industry. From ICT's through videoconference visits, patient training through telemedicine, diagnostic procedures that will also be transmitted through ICT processes along with home titling that can be adjusted every day in difficult cases, we believe it will be a new efficient and cost effective way to understand the management of these patients. At no time does this project attempt to diagnose all patients because, as with COPD, and given its prevalence, in the SAHS all health links must be involved from family medicine, nurses, general specialists, professionals with expertise in sleep pathology and companies providing respiratory therapies. The great advantage of ICTs will also be that, in the near future, it will allow networking between the different care links.

**CLINICAL IMPACT**:

SAHS affects 4-8% of the adult population. More than 20% of the population has an abnormal number of respiratory events during the night. This project is aimed at developing a new form of management of subjects with suspected SAHS and sleep pathology in general. Since SAHS is much better known in social media and among health professionals, this will involve a greater number of patients. At present in addition the typical patients are probably no longer the most numerous or in the near future will no longer be the SAHS coexists with many serious diseases such as heart failure along with other sleep disorders or rib cage. sophisticated CPAP equipment or ventilation and other sleep disorders to be detected. All these aspects make many patients clinically difficult to manage. Consequently, they will require more specifically trained professionals to care for them and hence we hospital professionals must develop systems to care for these more complex patients, ideally outside the hospital setting. There is no doubt that ICTs are going to be useful tools.

**PROMOTION OF SYNERGIES** **AND PROMOTION** OF TALENT IN THE SNS. This is a multidisciplinary project that requires the contribution of professionals working in outpatient clinics, in hospitals as well as experts in statistics, technology and above all in cost effectiveness. It should be noted that the PI of the project is the coordinator of the CIBERES Sleep Apnea programme. The project will continue to promote synergies, not only between technological, statistical or cost-effectiveness groups but also, as has already been initiated in the case of Impulse of Talent. This project, like the previous projects of the PI, will enhance the talent of the youngest members of the team, in what is expected to be a fundamental step in their scientific/technical career in the field of biomedicine. The group already tutors young researchers already included (Mireia Dalmases and Marta Torres). Dr Isaac Almendros who was part of the research group, and is currently working at the University of Chicago


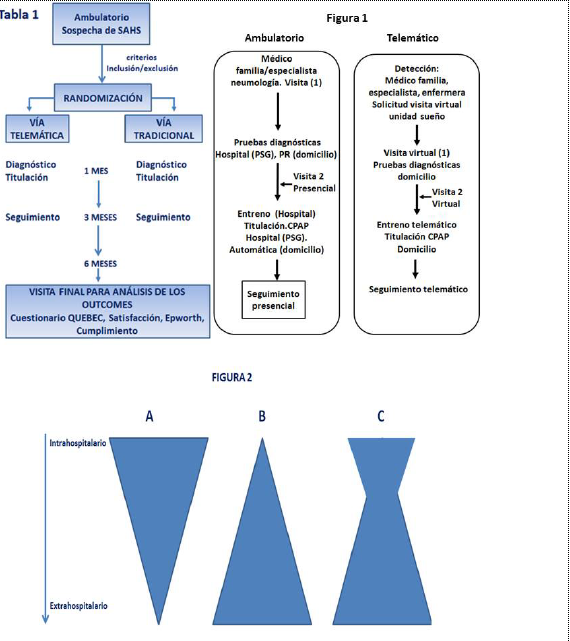

Supplement: S2 File — (DOCX) [file pone.0224069.s010.docx]
