# Supplementary material for: Comprehensive management of obstructive sleep apnea by telemedicine: Clinical improvement and cost-effectiveness of a Virtual Sleep Unit. A randomized controlled trial
Source: PLoS One. 2019 Oct 24;14(10):e0224069. doi: 10.1371/journal.pone.0224069 (PMC6812794; doi:10.1371/journal.pone.0224069)
Supplement: S3 File — (PDF) [file pone.0224069.s011.pdf]

CONVOCATORIA DE AYUDAS DE PROYECTOS DE INVESTIGACIÓN EN SALUD
MEMORIA DE SOLICITUD

Expediente Nº

TITULO: **Aplicabilidad, eficacia y coste efectividad de un modelo de gestión para el síndrome de apneas durante el sueño (SAHS) basado la Tecnología de la Información y Comunicación (TIC's)**

INVESTIGADOR/A PRINCIPAL: Josep M Montserrat

TIPO DE PROYECTO: ◉ **INDIVIDUAL** ○ **COORDINADO** ○ **MULTICÉNTRICO**

NOMBRE DEL IP COORDINADOR:
(Cumplimentar sólo en caso de proyectos coordinados)

DURACION: ☒ **3 AÑOS**

RESUMEN (Objetivos y Metodología del Proyecto) **(Máximo 250 palabras)**

Este proyecto desarrolla una nueva forma de gestión de los sujetos con sospecha de padecer un SAHS y de la patología del sueño en general mediante la aplicación de las TIC's. Es la respuesta a la mayor detección de pacientes que implicará mayor número de casos típicos con problemas y también casos complejos o en poblaciones "difíciles" que requerirán especialistas de patología del sueño que deberán gestionar el SAHS de un modo coste efectivo. **Objetivos.** Primario: 1. Análisis de eficacia y coste efectividad de dos programas de gestión del SAHS: el clásico hospitalario frente a otro telemático basado en la utilización de las TIC's. Se toma como variable principal el cuestionario de Quebec y en los casos de CPAP el cumplimiento. Secundarios: Otras variables Cuestionarios: Epworth, EuroQol-5D, número efectos secundarios, y cuestionario de satisfacción en ambos grupos. **Diseño:** prospectivo, aleatorizado, controlado, abierto en 202 pacientes (101 por rama). El seguimiento será de 6 meses con 4 evaluaciones. **Análisis estadístico:** Eficacia: se comparará el cambio de la escala Quebec antes y después de la intervención entre ambos brazos mediante análisis de la ANCOVA (premisa de mejoría de 2 puntos en el grupo telemático). Las variables secundarias se analizarán mediante las diferencias en medias independientes (o equivalente no paramétrico) o Chi2 para variables dicotómicas. Coste-efectividad**:** los costes generados por uno u otro programa se evaluarán frente a la efectividad de las variables principales mediante el cálculo del ICER siguiendo técnicas Bayesianas.

TITLE: Applicability, efficacy and cost effectiveness of the sleep apnea hypopnea syndrome (SAHS) management by using the technology based on information and communication (TIC's)

ABSTRACT (Objectives and Methodology of the project)

This project develops a new form of management of subjects with suspected SAHS and different sleep disorders by applying TIC's. It is the response to the increased detection of patients with SAHS. If there is more cases will be more typical cases with problems as well as complex patients that require sleep specialist. At present this patients must be managed in a cost-effective way. TIC's could be a response. **Objectives**. 1) Analysis of efficacy and cost effectiveness of two management programs of SAHS. The classic hospital one versus another based on work using ICT. The main variable is the Quebec questionnaire and in cases of CPAP the compliance. as the analysis of other variables EuroQol-5D, Epworth, the number side effects, and satisfaction in both groups. **Design**: Prospective, randomized, controlled, open, parallel in 202 patients (101 per arm). Monitoring will be 6 months and 4 assessments. Statistical analysis: Effectiveness: Changes in the Quebec scale is compared before and after intervention between the two arms using analysis of ANCOVA (premise 2-point improvement in the telematics group). Secondary variables were analyzed using independent differences in means (or nonparametric equivalent) or Chi2 for dichotomous variables. Cost-effectiveness: the costs generated by either program will be measured against the effectiveness of the main variables by calculating the ICER following Bayesian techniques

Expediente Nº	

INVESTIGADOR/A PRINCIPAL: Josep M Montserrat

MEMORIA DE SOLICITUD PROYECTO DE INVESTIGACIÓN EN SALUD
SECCIÓN ANTECEDENTES Y ESTADO ACTUAL DEL TEMA

Finalidad del proyecto, antecedentes y estado actual de los conocimientos científico-técnicos, grupos nacionales o internacionales que trabajan en la línea específica del proyecto o en líneas afines.
Citar las referencias en el apartado siguiente: Bibliografía más relevante. Máximo 3 páginas.

Este proyecto está encaminado a desarrollar una nueva forma de gestión de los sujetos con sospecha de padecer un síndrome de apneas-hipopneas durante el sueño (SAHS) y de la patología del sueño en general. En la práctica clínica estos pacientes pueden dividirse en 3 grandes grupos: A) Aquellos con un "pre-test" alto (con signos/síntomas típicos sin comorbilidades); B) Pacientes con sospecha de SAHS pero con patología significativa asociada (fibromialgia, insomnio u otra patología del sueño) que puede dificultar su valoración y requerir técnicas especiales; y C) Sujetos con bajo pre-test, o poblaciones especiales (edad avanzada, embarazo, ictus, cardiopatía severa etc.) que no siempre son fáciles de orientar. Estos dos últimos grupos consultan frecuentemente en diferentes niveles asistenciales requiriendo muchas pruebas y su diagnóstico acaba haciéndose siempre en unidades de tercer nivel. El grupo A es más fácil de atender y sin duda es imprescindible que su gestión vaya desarrollándose fuera de los hospitales o al menos de los de referencia. Sin embargo no es así los grupos B y C. Dado que el SAHS ha penetrado en la sociedad y entre los profesionales de la salud, existe en la actualidad un mayor número de pacientes diagnosticados de todos los grupos mencionados. Ello comporta o va a comportar una serie de problemas, por ejemplo, en el tratamiento con CPAP de los pacientes típicos o alto pre-test. En este caso, aunque la proporción de sujetos con problemas durante el tratamiento con CPAP (bajo cumplimiento, problemas locales, persistencia de la somnolencia, etc) será igual, el número total de pacientes va a ser mayor. Además, como ya ha sido mencionado, el SAHS coexistirá con diversas enfermedades asociadas tales como la patología cardíaca severa o la fibromialgia, o en poblaciones diferentes como la de edad avanzada, embarazadas, niños, etc. Todos los aspectos citados hacen que, desde el punto de vista clínico, muchos pacientes sean difíciles de gestionar y, en consecuencia, requieran profesionales más específicamente preparados para atenderlos. Con la idea de que cuando una enfermedad es frecuente todos los niveles asistenciales deben de estar implicados, este proyecto pretende desarrollar una nueva forma de atender y gestionar a estos pacientes con un alto nivel de calidad, un adecuado cumplimiento y que el procedimiento sea coste-eficaz. Para ello, se propone trabajar mediante la aplicación de las TIC´s, con visitas virtuales y estudios en domicilio básicamente con técnicas de telemedicina como hilo conductor asistencial. Este proyecto es fruto de la trayectoria del propio grupo en querer avanzar continuamente en la forma de diagnosticar a los pacientes con SAHS que se inició en 1997 con la descripción de la utilidad de los sistemas simplificados para diagnosticar y tratar (Parra et al. ERJ 1997, Lloberes et al AJRCCM 1996). Después, utilizando este sistema durante varios años en tres estudios multicéntricos publicados por el grupo español de sueño, se han consolidado las propuestas previas (Masa et al AJRCCM 2011, Thorax 2011, AJRCCM 2004). Recientemente hemos establecido la utilidad de la telemedicina en estudios piloto (Isetta et al. EJMR 2013). De este modo, y como resultado de toda la experiencia previa, creemos estar en condiciones de plantear un proyecto encaminado a una transferencia real en la práctica diaria.

El SAHS es muy prevalente. Se define como un cuadro de síntomas que alteran la calidad de vida junto a trastornos metabólicos, neurológicos y cardiovasculares (1-6) secundarios a un número anormal de episodios de obstrucción de la vía aérea superior. Está asociado a los accidentes de tráfico (7) y se relaciona con un exceso de mortalidad (8). Los pacientes no diagnosticados consumen más recursos sanitarios (9) y se le considera un problema de salud pública de primera magnitud (1). El incremento de su conocimiento ha producido un aumento en la demanda de consultas y la necesidad de realizar más estudios diagnósticos, sin embargo, no han ido acompañados en muchos casos de nuevas estrategias de diagnóstico, tratamiento y seguimiento de la enfermedad. Además el hecho de que el SAHS se asocie

frecuentemente a otras patologías y que sea considerado una enfermedad crónica (10) hace que sea necesario un nuevo modelo de enfocar su gestión para que sea más viable, coste-efectiva y sobre todo eficaz. Se plantean dos puntos como conceptos generales a considerar:

1) **Niveles asistenciales**. Al igual que ocurre con la EPOC, y dada dada su prevalencia, en el SAHS todos los eslabones sanitarios deben de estar implicados desde la medicina de familia, enfermeras, especialistas generales, profesionales expertos en patología del sueño hasta las empresas proveedoras de terapias respiratorias. Es decir, trabajo en red. Nuestro grupo tiene experiencia en este sentido pues ya en el 2007 iniciamos este tipo de estudios. Mostramos como los neumólogos generales (con rotación en sueño durante la residencia) trabajando en hospitales no de referencia o en centros de primaria en conexión con la unidad del sueño hospitalaria, manejan adecuadamente muchos pacientes con SAHS y sobre todo desde los ambulatorios se solicitan las pruebas correspondientes de un modo adecuado (polisomnografía convencional o respiratoria -domicilio y hospital- y oximetría) (11,12). Sin duda esta es una parte muy importante del manejo de los pacientes con alto pre-test.

2) **Telemedicina** o la aplicación de las nuevas tecnologías de información y comunicación (TIC's) a la asistencia clínica. En los últimos años la tecnología en medicina se aplica ampliamente y se ha visto potenciada por la incorporación de innovaciones efectivas surgidas de diversa áreas como la bioingeniería. Una de las pocas excepciones en este rápido proceso de aplicación o adaptación en los avances tecnológicos se encuentra en la medicina asistencial, la medicina del día a día. Su implementación y penetración en la práctica clínica es mucho menor que en otros campos científico-profesionales o incluso en nuestra vida cotidiana (13). El déficit asistencial mediante los sistemas de telemedicina no deja de ser sorprendente sobre todo en la patología del sueño, especialmente en el campo del SAHS en donde son precisos de un modo urgente nuevos protocolos para paliar su grave crisis asistencial: infra-diagnóstico, comorbilidad y largas listas de espera. Se han puesto en marcha algunas iniciativas, aunque pocas, tanto en EE.UU. como en Europa y también en España, (14) aunque muy tímidamente. Este proyecto pretende aplicar de un modo rutinario el uso las TIC´s en la práctica clínica de pacientes con sospecha de SAHS. Un punto muy significativo es el hecho de que es necesario adecuar la telemedicina al tipo de pacientes, pues su aplicación a ciertas poblaciones puede no resultar eficaz, sino todo lo contrario. Recientemente, se ha descrito mayor morbilidad o incluso mortalidad en pacientes multimórbidos de edad avanzada o con EPOC (Takahashi et al. Arch Intern Medicine 2012, Hallet et al. Ann of Internal Medicine 2012). Por nuestra experiencia en los trabajos piloto realizados recientemente, los pacientes con sospecha de SAHS pueden beneficiarse mucho de las TIC's. Para gestionar totalmente el procedimiento de diagnóstico, tratamiento y seguimiento de una forma innovadora (Figura 1) hemos realizado una serie de estudios piloto que cubren desde el entreno al seguimiento del tratamiento, siendo estas experiencias muy positivas, lo que nos permite afrontar un proceso global (15).

En la actualidad la mayoría de pacientes son seguidos en los centros de referencia (Figura 2-A) y obviamente en el contexto actual no es lógico. Es de prever que los pacientes no complejos se manejen fuera del hospital (Figura 2-B). Sin embargo, los casos complejos, tales como mal cumplimiento, obesidad mórbida, tratamientos quirúrgicos, Cheyne Stokes, servoventilación, edad avanzada o patologías asociadas severas, entre otros, deben ser atendidos en las unidades de sueño, y es de esperar que en un futuro su número pueda incrementarse notablemente (Figura 2-C) y de ahí que los profesionales de los hospitales debamos adaptarnos a esta realidad cambiante y desarrollar sistemas para atender a este tipo de pacientes, idealmente fuera del ámbito hospitalario para optimizar el coste efectividad. Las TIC's son una opción ya que permiten el uso de: a) técnicas diagnósticas apropiadas (PSG extrahospitalaria), b) estudios de varios días como recientemente hemos publicado -tipo Holter-) (16) y c) teleconsultas (Figura 1). Todo ello gestionado por

especialistas en sueño (16) pues hemos comprobado que es una opción muy importante pues facilita las consultas que requieren menos tiempo, evita desplazamientos a los pacientes (pérdidas de horas de trabajo o que su residencia sea lejana o que su condición le impida desplazarse con facilidad). Además, las visitas puntuales van a mejorar el cumplimiento (15).

Como resumen cabe decir que para instalarse positivamente en esta situación tan difícil y cambiante como la actual se deben desarrollar diferentes estrategias de actuación. El trabajo en red es una opción, las técnicas de telemedicina también y la combinación de ambas va a ser probablemente la forma óptima para llegar a una medicina personalizada y coste-efectiva en donde intervengan todos los eslabones asistenciales. Nuestra experiencia nos permite acometer este amplio proyecto que pretende gestionar todo el proceso de atención al SAHS de una forma diferente y que se adecue a la realidad en parte relacionada con lo que se conoce como las 4 P de la medicina: 1. Personalizada (depende de caracteres del genoma) 2. Predictiva a partir de la información individual que puede establecer predicciones 3. Preventiva, a partir de las predicciones implementar medidas preventivas y 4. Participativa por parte del paciente. Para el desarrollo de este proyecto se va a comparar la eficacia y coste-efectividad entre un sistema tradicional y otro que utiliza fundamentalmente las TIC's (Tabla 1). Para realizarlo se podrían elegir dos opciones: 1) un estudio solo sobre la población "difícil" (pero en este caso no se conocería el comportamiento del grupo alto pre-test que podría ser muy diferente), o 2) que es la que hemos elegido, plantear el estudio de todo tipo de pacientes, sin valorar sus características iniciales. Creemos que esta última es mejor pues será más real y sobre todo que en el análisis posterior ya se analizarán los diversos grupos mencionados.

Expediente Nº

INVESTIGADOR/A PRINCIPAL: Josep M Montserrat

MEMORIA DE SOLICITUD PROYECTO DE INVESTIGACIÓN EN SALUD
SECCIÓN ANTECEDENTES Y ESTADO ACTUAL DEL TEMA

Citar las referencias incluidas en el apartado anterior: Antecedentes y Estado actual. **Máximo 1 página.**

1.Durán-Cantolla et al. Documento de consenso nacional sobre el SAHS. Arch Bronconumol 2005; 41:1-110.

2.Durán J et al. Obstructive sleep apnea-hypopnea and related clinical features in a population-based sample of subjects aged 30 to 70 years. Am J Respir Crit Care Med 2001; 163:685-9.

3.Baldwing et al. The association of sep-disordered breathing and sleep symptoms with quality of life in the Sleep Heart Healt Study. Sleep 2001; 24:96-105.

4.Martínez-García MA et al. Effect of CPAP on blood pressure in patients with obstructive sleep apnea and resistant hypertension: the HIPARCO trial. JAMA. 2013; 11; 310:2407-15.

5.Somers VK et al. Sleep apnea and cardiovascular disease. Circulation. 2008; 118:1080-111.

6.Yaggi HK et al. SAHS a a risk factor for stroke and death. NEJM 2005; 353:2034-41.

7.Terán-Santos J et al. The association between SAHS and the risk of traffic accidents. NEJM 1999; 340:847 -51.

8.Young T et al. Sleep-disordered-Breathing and mortality; Eigtheen-year follow-up of Wisconsin Sleep Cohort. Sleep 2008;31:1071-8.

9.Diaz K, et al. Obstructive sleep apnea is associated with higher healthcare utilization in elderly patients. Ann Thorac Med. 2014; 9:92-8.

10.Heatley EM et al Obstructive sleep apnoea in adults: a common chronic condition in need of a comprehensive chronic condition management approach. Sleep Med Rev. 2013; 17:349

11.Hernández L, et al.Management of sleep apnea: concordance between nonreference and reference centers. Chest. 2007;132:1853-7.

12.Chamorro N et al. An integrated model involving sleep units and primary care for the diagnosis of sleep apnoea Eur Respir J. 2013; 42:1151-4

13.Ramon Farre. Telemedicina y enfermedades respiratorias durante el sueño: perspectivas de futuro. ArchBronconeumol.2009;45(3):105 -106

14.Coma-Del-Corral MJ et al. Reliability of telemedicine in the diagnosis and treatment of sleep apnea syndrome. Telemed J E Health. 2013; 19:7-12

15.Isetta V et al. Telemedicine-based approach for obstructive sleep apnea management: building Evidence. Interact J Med Res. 2014; 19;3(1):e6. doi: 10.2196/ijmr.3060.

16.Guerrero A, et al. Management of Sleep Apnea without high pre-test probability or with comorbidities by three nights of portable sleep monitoring. Sleep August 2014

17.Negrín MA et al. A fully Bayesian cost-effectiveness analysis using conditionally specified prior distributions. In Health Care Costs: Causes, Effects and Control**, 2008.** pp. 1-17 (Chapter 7). Martel,

Expediente Nº

INVESTIGADOR/A PRINCIPAL: Josep M Montserrat

MEMORIA DE SOLICITUD PROYECTO DE INVESTIGACIÓN EN SALUD
SECCIÓN HIPÓTESIS Y OBJETIVOS

(Ajústese al espacio disponible)

HIPÓTESIS

- La eficacia del manejo del SAHS utilizando un trabajo basado en TIC's será superior a la del que se utiliza en la actualidad en el marco hospitalario que utiliza tanto poligrafía respiratoria o PSG, incluyendo todo tipo de pacientes desde los de alta probabilidad a los más complejos.
- El coste-efectividad del manejo del SAHS utilizando un trabajo basado en TIC's es superior al que se utiliza en la actualidad en el marco hospitalario que utiliza tanto poligrafía respiratoria o PSG, incluyendo todo tipo de pacientes desde los de alta posibilidad a los más complejos.
- Un óptimo coste-efectividad se producirá especialmente en los pacientes complejos. Estos pacientes contactarán directamente al centro de referencia el cual a través del uso de las TIC simplificara el proceso. Este mismo grupo de pacientes cuando siguen el proceso habitual requieren varias visitas en diversos niveles asistenciales y siempre acaban diagnosticándose en los centros de referencia.
- La transferencia del proceso telemático será posible y podrá ser incluido rápidamente en la rutina asistencial.

OBJETIVOS

Primarios:

- Evaluación de dos programas de gestión del SAHS, el clásico que se utiliza en la actualidad frente a otro basado en trabajo utilizando las TIC's tomando como variable principal el cuestionario de Quebec y en los casos con CPAP el cumplimiento del tratamiento.
- Análisis del coste-efectividad de los programas antes mencionados valorando como variable principal el cuestionario Quebec y en los casos de CPAP el cumplimiento del tratamiento.
- Eficacia del programa basado fundamentalmente en tecnología entre el grupo de pacientes complejos (Grupos 2 y 3 descritos inicialmente) y los alta posibilidad de padecer un SAHS (Grupo 1).

Secundarios:

Evaluación de los programas mencionados mediante el análisis suplementario de las siguientes variables:

1. Cuestionarios: Epworth y EuroQol-5D
2. Número de complicaciones o efectos secundarios
3. Cuestionarios de satisfacción de los pacientes

Expediente Nº	

INVESTIGADOR/A PRINCIPAL: Josep M Montserrat

MEMORIA DE SOLICITUD PROYECTO DE INVESTIGACIÓN EN SALUD
SECCIÓN PROYECTOS COORDINADOS

En caso de Proyectos Coordinados, el COORDINADOR deberá indicar:
- Objetivos globales del proyecto coordinado, la necesidad de dicha coordinación y el valor añadido que se espera obtener de la misma.
- Objetivos específicos de cada subproyecto (deben están recogidos además en la memoria de cada subproyecto)
- Interacción entre los distintos objetivos, actividades y subproyectos.
- Los mecanismos de coordinación previstos para la eficaz ejecución del proyecto. **Máximo 3 páginas.**

Expediente Nº

INVESTIGADOR/A PRINCIPAL: Josep M Montserrat

MEMORIA DE SOLICITUD PROYECTO DE INVESTIGACIÓN EN SALUD
SECCIÓN METODOLOGÍA
Diseño, sujetos de estudio, variables, recogida y análisis de datos y limitaciones del estudio. Máximo 3 páginas.

DISEÑO Y SUJETOS DEL ESTUDIO (ver figuras al final)**.** Estudio experimental, aleatorizado, abierto de grupos paralelos de pacientes en el que se realizará un análisis de eficacia del tratamiento y coste-efectividad. Se incluirán de manera consecutiva a todos los pacientes con sospecha diagnóstica de SAHS que pertenezcan a los centros de atención primaria (CAP) del área Esquerra del Eixample de Barcelona que corresponde al área de salud del Hospital Clínic de Barcelona. Se excluirán a aquellos pacientes con: a) incapacidad psico-física para realizar cuestionarios; b) dificultad para utilizar la tecnología de comunicaciones (smartphone), c) embarazo, d) la no obtención del consentimiento informado, e) somnolencia grave o invalidante o f) enfermedades inestables. Los pacientes serán aleatorizados, siguiendo una secuencia generada por una base de datos, en dos grupos (Tabla 1):

Grupo 1: Pacientes que siguen el procedimiento habitual (hospitalario), o sea después de la visita del médico de primaria a través del especialista de neumología extrahospitalario que tomará las decisiones oportunas y si procede se remite al hospital para hacer una prueba del sueño, titulación si procede y seguimiento durante 6-12 meses en el hospital (normativa SEPAR).

Grupo 2: seguimiento mediante telemedicina a cargo del centro de referencia: este grupo de pacientes serán detectados en el ambulatorio de la misma manera que el grupo 1 pero realizarán el diagnóstico, educación, tratamiento y seguimiento mediante un procedimiento telemático totalmente extrahospitalario realizado por médicos especialistas en patología del sueño (Figura 1) basado en las TIC's hasta que sea remitido si procede a su lugar de origen. Para minimizar los posibles sesgos de información derivados del procedimiento abierto, el profesional encargado de la introducción y valoración de los cuestionarios así como de otras variables principales objetivas (medición del cumplimiento de la CPAP) desconocerá el grupo de pertenencia del paciente.

ESQUEMA DE TRABAJO:

Variables

En los pacientes que hayan superado los criterios de inclusión/exclusión se recogerán los siguientes datos:
1) Datos antropométricos y clínicos relacionados con el SAHS. Síntomas relacionados con otras patologías del sueño. Antecedentes. Exploración física específicamente ORL maxilofacial. Tensión arterial. 2) Cumplimiento del tratamiento si se utiliza CPAP (lectura del contador y el tiempo óptimo de presión).
Dadas las características del proyecto y con el objeto de simplificar el protocolo que, por sus características debe de ser simple, se recogerán solo algunas variables suplementarias a las habituales, concretamente: cuestionario Quebec (Lacasse Y Thorax 2004), traduccio españolMedicina Clinica Merrejon 2012), escala de somnolencia de Epworth (Johns MW Sleep 1991) y EuroQol-5D (Eurocold group; health Policy 1990). Ademas escala de satisfacción y efectos secundarios. (Autoadministrados con 15 minutos de tiempo para su realización)

Procedimentos: Estudios de sueño y titulación de CPAP. 1)PSG o Poligrafía respiratoria (PR) hospitalaria: Se realizarán las técnica y análisis según normativas de AASM 2012-SEPAR 2011. **2) PSG o PR domicilio**. La técnica y análisis se ajustarán también a las normativas estandarizadas mencionadas. El grupo de enfermería de atención domiciliaria a los pacientes crónicos también entregará los equipos. En este caso los datos se transmitirán al hospital por vía telemática. Para la titulación de CPAP se seguirá el mismo procedimiento excepto que la empresa

suministrará el equipo automático para titulación, el entreno se hará desde el hospital vía telemática y en caso de ser una titulación difícil se ajustará durante varios días según resultados observados telematricamente. En el brazo de tratamiento habitual los profesionales de los centros tradicionales seguirán los procesos ya establecidos. Se considerarán estudios válidos para PSG o PR aquellos aquellos que cumplan las normativas del grupo español de sueño. En cuanto al tratamiento se seguirán las normativas SEPAR (Lloberes et al. 2011).

Seguimiento: Los pacientes serán evaluados según normativas generales de SEPAR (inicio, a los 1, 3 meses, y a los 6 meses) y sobre todo dependiendo del criterio médico. Obviamente se valorarán las visitas extra realizadas. Se recogerán los cuestionarios citados y en el caso de CPAP el cumplimento al inicio y al final (no se recoge cumplimiento de CPAP en los meses intermedios porque un grupo es telemático).

Justificación del tamaño de muestra. La variable principal de estudio es el cambio en la puntuación de la dimensión del cuestionario Quebec que requiera una "n" mas alta. Se estima que para un error alfa de 0.05, beta de 0.20 y una diferencia clínicamente relevante (cambio en la puntuación total ≥2 puntos en el cuestionario) entre los dos grupos de estudio, se precisan un mínimo de 101 pacientes por brazo de aleatorización (202 pacientes en total). Se estimado el 20% de pérdidas (entre los que se pierdan y los que no toleren la CPAP), según los datos publicados acerca del grado de cumplimiento de la CPAP en España que se estima entre el 75-80%.

Estadística:

Objetivos principales: Tras una depuración de los datos recogidos las variables del estudio se tabularán en una base de datos. Para la identificación de los pacientes se asignará un código específico único. La clave de conexión entre el código y el sujeto estará en manos del IP que asumirá toda la responsabilidad de protección y cuidado de los datos. La construcción de la base de datos, valoración de cuestionarios, entrada de datos a la base de datos y análisis de los resultados será realizados por personal ajenos a la investigación. La construcción de la base de datos y análisis de los datos serán realizados por profesionales informáticos y estadísticos respectivamente. Estudio univariado: En el caso de las variables cualitativas se determinará su frecuencia y porcentaje válido. Se tendrán en cuenta los valores perdidos para dar los resultados. Para las variables cuantitativas se incluirán medidas de tendencia central, medidas de posición y de dispersión.

Valoración de la eficacia. El análisis estadístico se basará en el principio de intención de tratar. La comparación en los cambios en la puntuación del cuestionario QUEBEC (6 meses vs. Basal) entre ambos grupos se realizará a partir del test de ANCOVA obteniendo las medias de puntuaciones a los 6 meses para cada grupo ajustadas por los valores basales y otras variables de confusión como pueden ser el IMC, la edad o la gravedad del SAHS. Este test se realizará bajo el supuesto de "mínimos cuadrados". Se realizará sub-análisis con los pacientes más complejos en el que se replicará el mismo análisis. Se realizará también un análisis por protocolo en el caso de los pacientes con CPAP según el cumplimento sea inferior o no a 4 h/noche. Para los pacientes sin la última visita de seguimiento, y por lo tanto, únicamente con información basal, se imputará el mismo valor basal a la visita de las 24 semanas por tratarse de la opción más conservadora y en contra de la hipótesis del potencial beneficio de la intervención con CPAP. Para todos los test se establecerá un diferencia significativa <0,05 (se usará SPSS versión 20).

Coste-efectividad: Los costes generados por uno y otro protocolo se evaluarán frente a la efectividad de la variable principal Quebec, también como secundarias (Epworth y EuroQol-5D), mediante técnicas de análisis coste-efectividad Bayesianas. Las técnicas Bayesianas se presentan especialmente adecuadas para este proyecto donde la experiencia del decisor podrá ser incorporado al estudio mediante una distribución de probabilidad a priori que refleje adecuadamente sus juicios sobre las variables estudiadas. Además obviaremos los supuestos usuales de costes normales y podremos utilizar costes asimétricos más cercanos a la práctica real. Esto nos llevará a la utilización de las técnicas Markov Chain Monte Carlo (MCMC) para la simulación de las medidas de interés: la ratio coste-efectividad de cada tratamiento, la ratio coste-efectividad incremental (ICER), el plano coste-efectividad, el

beneficio neto de cada tratamiento, el beneficio neto incremental (NB) y la curva de aceptabilidad coste-efectividad. Cada una de estas medidas tiene su interpretación natural en términos de probabilidad y de disposición a pagar del decisor frente a un incremento en el estado de salud del paciente, haciéndolas especialmente atractivas por su aplicabilidad práctica. El proceso estadístico-matemático no es sencillo, pero en este proyecto, diseñaremos un conjunto de códigos de fácil implementación informática de tal forma que el decisor obtenga de forma fácil y sencilla estas medidas de interés para su posterior toma de decisiones (17).
Objetivos secundarios: Comparación entre ambos brazos de los cuestionarios citados (Epworth, EuroQol-5D, Satisfacción y número de efectos secundarios). Además al disponer de otras variables de interés recogidas a cada paciente, estas variables podrán actuar a modo de covariables que ayuden a minimizar la incertidumbre en la estimación de la efectividad y el coste incremental. Proponemos por tanto una serie de objetivos secundarios ligados a la metodología bayesiana propuesta: 1) desarrollar un coste-efectividad para más de una medida de efectividad, proponiendo además medidas de toma de decisiones claras y de fácil interpretación y 2) Desarrollar un modelo BMA (Bayesian Model Averaging) para la estimación del coste-efectividad. La solución BMA se obtiene como una media de los coeficientes obtenidos por todas las posibles combinaciones de las covariables ponderadas por la probabilidad a posteriori de cada modelo. Esto implica la selección de qué variables poseen capacidad predictiva ya que la elección de un único modelo entre todos los posibles no considera la incertidumbre asociada a la elección del modelo correcto. Este objetivo nos permitirá una innovación en este tipo de estudio ya que seleccionaremos de forma automática las variables relevantes mediante la selección bayesiana de covariables (17).
Logística: a) Protocolo: basado en la memoria presente; b) Cuaderno de procedimientos: explica paso a paso el desarrollo del estudio y contiene todos los cuestionarios, pruebas y la forma de llevarlos a cabo para la estandarización, c) Cuaderno de recogida de datos que contiene las variables que han de recogerse distribuidas en las visitas. El orden y formulario es el mismo que el de la base de datos; d) Base de datos con dominio perteneciente al centro solicitante.
Aspectos éticos: El estudio se llevará a cabo de acuerdo con las directrices y los principios de la Declaración de Helsinki y será aprobado por el Comité de Ética e Investigación Clínica. Todos los sujetos del estudio proporcionarán por escrito el consentimiento informado antes de participar. El procedimiento de garantía de calidad será permanente (gestión de datos, procedimientos de seguridad con datos son suficientes, eficaces y bien documentados).
Ventajas y limitaciones: La ventaja fundamental de este protocolo es que se corresponde con la realidad. Muchas investigaciones comparan estudios en centros de referencia con solo PSG vs un sistema domiciliario. Es obvio que siempre el análisis de coste-efectividad va a ser favorable al domiciliario pues de los pacientes que acuden al hospital, en la actualidad más de un 60%, se resuelven con poligrafía respiratoria. Además, una vez puesto en marcha el protocolo la intervención del gestor de proyecto va a ser de observación precisa sin que intervenga en ningún momento en los diferentes eslabones. Por otra parte, la elección de una metodología bayesiana para el análisis coste-efectividad nos presenta ventajas comparativas con las técnicas clásicas basadas en la aproximación normal: utilización de costes asimétricos, utilización de efectividades continuas y/o discretas, interpretabilidad natural de las medidas de interés en términos de probabilidad, etc.
Limitaciones o problemas: a) Volumen de pacientes. Aunque es elevado no esperamos tener problemas en la inclusión pues entre los diversos ambulatorios que trabajan en nuestra aérea se recibe cada año 180 pacientes. b) Conocimiento de informática. Para poder participar es preciso saber utilizar técnicas básicas de internet, ordenador, smartphobe y/o tablet. Estimamos que un 25% de la población en nuestro medio no lo utiliza. En este sentido este punto no invalida en absoluto el trabajo pues la tendencia es claramente hacia la reducción de esta porcentaje a valores mínimos. c) Seguimiento: para mantener “vivo” el proyecto se ha previsto una evaluación externa independiente cada 6 meses así como reuniones con los médicos del ambulatorio.

Expediente Nº

INVESTIGADOR/A PRINCIPAL: Josep M Montserrat

MEMORIA DE SOLICITUD PROYECTO DE INVESTIGACIÓN EN SALUD
SECCIÓN PLAN DE TRABAJO

Etapas de desarrollo y distribución de las tareas de todo el equipo investigador, y las asignaciones previstas para el personal técnico que se solicita. Indicar además el lugar/centro de realización del proyecto.

(Ajústese al espacio disponible)

4.2 ESQUEMA DE TRABAJO:

Primeros 6 meses. Hospital Clínic y los diversos colaboradores Poner en marcha todos los medios precisos: página web, registro para entrada electrónica, reuniones para definir bien los protocolos y la lectura de los estudios de sueño. Reuniones con los profesionales que colaborarán (expertos en estadística y coste efectividad).

Próximos 24 meses: Hospital Clínic/Ambulatorios. Captación de los pacientes que acuden a los ambulatorios. Es el tiempo estimado para la entrada de todos los pacientes y su seguimiento a cargo de los profesionales médicos del área de influencia del Hospital Clínic.

Ultimos 6 meses: Hospital Clínic. Análisis de los resultados con la ayuda de un estadístico con experiencia y expertos en coste efectividad y las primeras comunicaciones a congresos.

Expediente Nº

INVESTIGADOR/A PRINCIPAL: Josep M Montserrat

MEMORIA DE SOLICITUD PROYECTO DE INVESTIGACIÓN EN SALUD
SECCIÓN PLAN DE TRABAJO
(Ajústese al espacio disponible. Puede incorporar hasta un máximo de 8 líneas de Actividad/Tarea)

CRONOGRAMA

ACTIVIDAD / TAREA	PERSONA/S INVOLUCRADAS		MESES E	F	M	A	M	J	J	A	S	O	N	D
Reunión investigadores	Todos	1º Año	✓	✓										
		2º Año												
		3º Año												

ACTIVIDAD / TAREA	PERSONA/S INVOLUCRADAS		MESES E	F	M	A	M	J	J	A	S	O	N	D
Reclutamiento	Centro investigador	1º Año							✓	✓	✓	✓	✓	✓
		2º Año	✓	✓	✓	✓	✓	✓	✓	✓	✓	✓	✓	✓
		3º Año	✓	✓	✓	✓	✓	✓						

ACTIVIDAD / TAREA	PERSONA/S INVOLUCRADAS		MESES E	F	M	A	M	J	J	A	S	O	N	D
Analisis de datos y presentación a congresos	Centro investigador Especial. estadistica y coste-efectividad	1º Año												
		2º Año												
		3º Año							✓	✓	✓	✓	✓	✓

Expediente Nº

INVESTIGADOR/A PRINCIPAL: Josep M Montserrat

MEMORIA DE SOLICITUD PROYECTO DE INVESTIGACIÓN EN SALUD
SECCIÓN PLAN DE TRABAJO

Inserte (si lo desea) una imagen con un cronograma.

Tabla 1

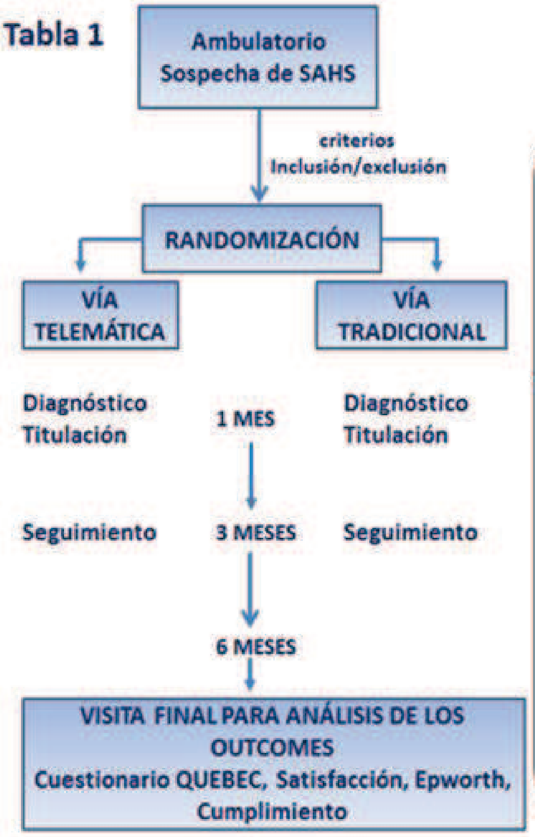

Figura 1

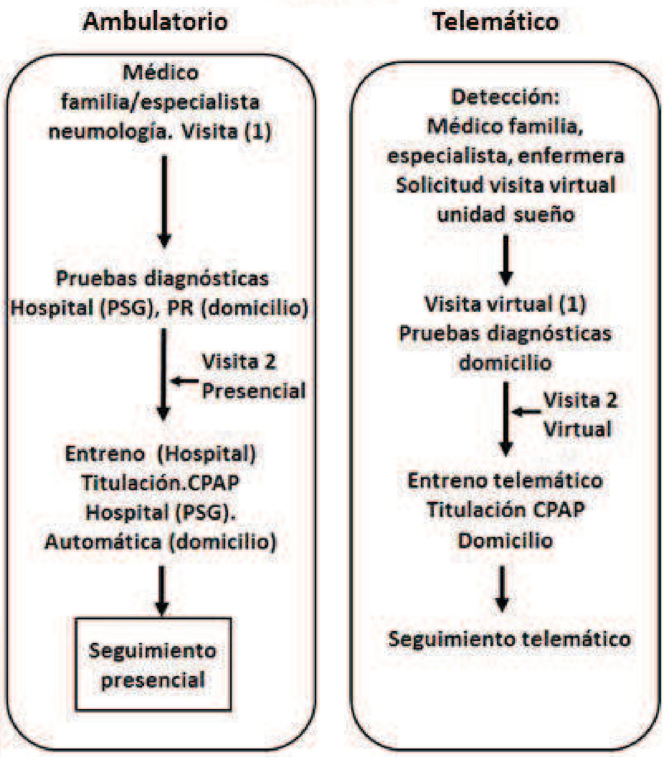

FIGURA 2

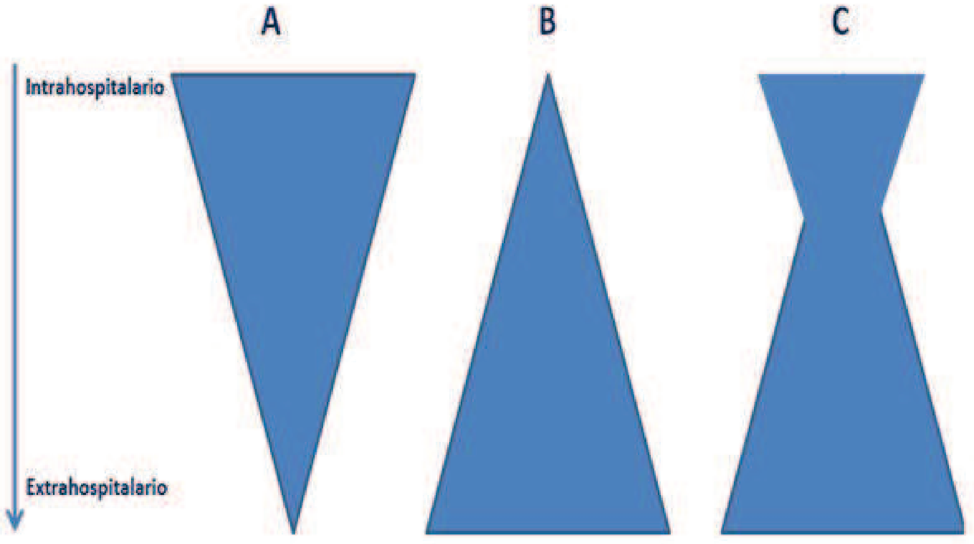

Expediente Nº

INVESTIGADOR/A PRINCIPAL: Josep M Montserrat

MEMORIA DE SOLICITUD PROYECTO DE INVESTIGACIÓN EN SALUD
SECCIÓN EXPERIENCIA DEL EQUIPO

Experiencia del equipo investigador sobre el tema **(Ajústese al espacio disponible)**

La unidad multidisciplinaria del Sueño del Hospital Clínic de Barcelona realiza una actividad asistencial prolífera en la cual contribuyen los siguientes servicios: Neumología, Neurología, Otorrinolaringología, Cirugía máxilo-facial, Dietética, Psicologia, Psiquiatría y Enfermeria. Desde 1990, como resultado de un acuerdo entre el Hospital Clínic y el Institut Català de la Salut (ICS), es Centro de Referencia para el diagnóstico de las alteraciones respiratorias relacionadas con el sueño. Ademas la unidad está dotada con todo el material necesario para realizar estudios del sueño (polisomnografías, poligrafías respiratorias, técnicos y secretaria). Anualmente se realizan 1500-1700 estudios. Respecto a la investigación, la unidad desarrolla su actividad en el marco del IDIBAPS y de la Facultad de Medicina de la Universidad de Barcelona. Tiene como objetivo prioritario el mejor conocimiento del SAHS. Los estudios realizados están orientados en dos vertientes diferentes: los clínicos y los más básicos o tecnológicos. Los estudios clínicos se llevan a cabo en colaboración con el grupo español de patología respiratoria del sueño (el solicitante de este proyecto es en la actualidad el coordinador).

Asimismo el grupo colabora otros grupos de la Universidad de Barcelona, especialmente en cuanto a los estudios tecnológicos y con modelos animales con miembros de la Unidad de Biofísica y Bioingeniería (Dr. R. Farré y Dr. D. Navajas). Al igual que en los aspectos clínicos, en investigación básica y tecnológica se han realizado varios estudios publicados en revistas de gran impacto (ver publicaciones currículum IP) y muy referenciados en la literatura médica.

Las líneas de trabajo han sido: 1) Estudios multicéntricos con el grupo español de patología del sueño, 2) Aspectos fisiológicos y consecuencias del SAHS (Eficacia de la CPAP) 2), Aspectos tecnológicos (desarrollo de un modelo mecánico para conocer el funcionamiento de las diversas CPAP automáticas, validación de la sonda nasal como método óptimo de medir flujo, aplicación de la técnica de oscilación forzada al sueño para estudios fisiopatológicos y 3) En los últimos años ya hemos iniciado el estudio de mecanismos a través de mediadores biológicos y modelos de rata y ratón para profundizar y dar un paso más en el estudio de la patología respiratoria del sueño a través de una visión de elementos básicos de la enfermedad.

En los últimos años como consecuencia de una reestructuración del área sanitaria un grupo de ambulatorios han pasado a ser gestionados conjuntamente con el Hospital Clínic (Área Esquerra del Eixample de Barcelona).

Respecto a los análisis de coste efectividad que es uno de los grandes “outcomes de este proyecto el grupo va a colaborar con el equipo del Prof Francisco Jose Vazquez Polo. Este grupo El grupo de investigación TEBADM está adscrito a la Universidad de Las Palmas de Gran Canaria tiene una amplia experiencia investigadora en el campo de la Estadística Bayesiana en general y del coste-efectividad (Economía de la Salud), en particular. El número y calidad de sus publicaciones avalan la capacidad del equipo de investigación para llevar a cabo este nuevo proyecto. A título de ejemplo y en ambos tópicos de investigación, en los últimos 5 años el grupo algunas publicaciones en revistas internacionales (todas ellas JCR) han sido: ASTIN Bulletin (2012, 2013), Bayesian Analysis (2009, 2010), Communications in Stistics: Theory and Methods (2011), Computational Statistics and Data Analysis (2010), European Journal of Health Economics (2013 aceptado), European Journal of Operational Research (2010, 2012, 2013). Respecto a los grupos nacionales e internacionales que trabajan en materias afines, destacan: (1) Los contactos directos con los grupos internacionales de primer nivel. El grupo trabaja en área de selección bayesiana de modelos (grupos radicados en las universidades italianas de Padova, Cagliari y Pavia, 2) respecto a proyectos competitivos obtenidos recientemente cabe citar: Técnicas Estadísticas Bayesianas para la Toma de Decisiones Económicas. Aportaciones y Aplicaciones a los siguientes campos: Estadística Actuarial, Análisis Coste-Efectividad para Datos Clínicos y Auditoría de Cuentas. Ministerio de Educación y Ciencia (SEJ2006-12685). 2006-09, finalmente (3) el grupo también trabaja en nuevas metodologías estadísticas para la toma de decisiones económicas en el ámbito de la economía de la salud (Consejería de Innovación, Ciencia y Empresa de la Junta de Andalucía mediante técnicas de inteligencia artificial.

Expediente Nº

INVESTIGADOR/A PRINCIPAL: Josep M Montserrat

MEMORIA DE SOLICITUD PROYECTO DE INVESTIGACIÓN EN SALUD
SECCIÓN MARCO ESTRATÉGICO

(Ajústese al espacio disponible)

1. Capacidad del proyecto de abordar los objetivos, prioridades enmarcadas en el reto Salud, Cambio Demográfico y Bienestar de la Estrategia Española de Ciencia y Tecnología y de Innovación.
2. Capacidad del proyecto de fomentar sinergias e impulsar el talento en el SNS.

IMPACTO TECNOLÓGICO. El proyecto plantea el desarrollo de un sistema con innovaciones en el manejo de pacientes utilizando tecnología que encaja en el campo de la investigación de transferencia actual. Estos avances tienen potencial interés comercial y son candidatos para la transferencia a la industria. De las TIC's a través de las visitas mediante videoconferencias, el entreno de los pacientes mediante telemedicina, los procedimientos diagnósticos que serán trasmitidos también mediante procesos TIC junto a las titulaciones domiciliarias que pueden ajustarse día a día en los casos difíciles, creemos que va a ser una nueva forma eficiente y coste efectiva de entender la gestión de estos pacientes. En ningún momento este proyecto pretende diagnosticar a todos los pacientes pues, al igual que ocurre con la EPOC, y dada su prevalencia, en el SAHS todos los eslabones sanitarios deben de estar implicados desde la medicina de familia, enfermeras, especialistas generales, profesionales expertos en patología del sueño y empresas proveedoras de terapias respiratorias. La gran ventaja de las TIC's también será que en un futuro cercano va a permitir precisamente el trabajo en red entre los diferentes eslabones asistenciales.

IMPACTO CLÍNICO: El SAHS afecta al 4-8% de la población adulta. Más de un 20% de la población tienen un número anormal de eventos respiratorios durante la noche. Este proyecto está encaminado a desarrollar una nueva forma de gestión de los sujetos con sospecha de SAHS y de la patología del sueño en general. Dado que el SAHS es mucho más conocido en los medios sociales y entre los profesionales de la salud, ello va a implicar un mayor número de pacientes. En la actualidad además los típicos pacientes probablemente ya no son los más numerosos o en un futuro próximo ya no lo serán el SAHS coexiste con muchas enfermedades graves como la insuficiencia cardíaca junto a otras patologías del sueño o la caja torácica. equipos sofisticados de CPAP o ventilación y otras enfermedades del sueño que se detectaran. Todos los aspectos citados hacen que desde el punto de vista clínico muchos pacientes sean difíciles de gestionar. En consecuencia, van a requerir profesionales más específicamente preparados para atenderlos y de ahí que los profesionales de los hospitales debamos desarrollar sistemas para atender a estos pacientes más complejos, idealmente fuera del ámbito hospitalario. Sin duda las TIC's van a ser instrumentos útiles.

FOMENTO DE SINERGIAS E IMPULSO DEL TALENTO EN EL SNS. Este es un proyecto multidisciplinar que requiere la aportación de profesionales que trabajan en los ambulatorios, en los hospitales así como expertos en estadística, en tecnología y sobre todo en coste efectividad. Es de destacar que el IP del proyecto es el coordinador del programa de Apnea del Sueño del CIBERES. El proyecto seguirá promoviendo sinergias, no solo entre grupos tecnológicos, estadísticos o de coste efectividad sino también, como ya se ha iniciado en el caso Impulso del talento. Este proyecto, como los anteriores del IP, potenciará el talento de los miembros más jóvenes del equipo, en lo que se espera que sea un paso fundamental en su carrera científico/técnica en el ámbito de la biomedicina. El grupo ya tutela investigadores jóvenes ya incluidos (Mireia Dalmases y Marta Torres). El Dr Isaac Almendros quien formó parte del grupo investigador, y actualmente se encuentra trabajando en la Universidad de Chicago.

Expediente Nº

INVESTIGADOR/A PRINCIPAL: Josep M Montserrat

MEMORIA DE SOLICITUD PROYECTO DE INVESTIGACIÓN EN SALUD
SECCIÓN MEDIOS DISPONIBLES

(Ajústese al espacio disponible)

Medios disponibles para la realización del proyecto

El grupo dispone de:

1. Un laboratorio del sueño completo para realizar PSG convencionales.
2. Equipamiento para realizar estudios domiciliarios y simplificados en el hospital.
3. Unas consultas externas especificas en donde se controlan mas de 2500 pacientes en la actualidad conjuntamente con la empresa proveedora de servicios.
4. Un grupo multidisciplinar de patología del sueño en donde colaboran: ORL, neurología, psiquiatría, psicología, enfermería y neumología.
5. Una unidad de consulta externa unificada para el grupo multidisciplinar.
6. Diversos ambulatorios que trabajan en el área del Hospital Clínic.
7. Ademas el grupo colabora con la unidad de Biofísica y Bioingeniería de la Facultad de Medicina de la Universidad de Barcelona (Dr. R. Farré, Dr. D. Navajas, Dra. V. Isetta).
8. Especialmente importante es la colaboración del grupo del Dr. Francisco José Vázquez Polo, del Departamento de Métodos Cuantitativos, Facultad de Ciencias Económicas y Empresariales de la Universidad de Las Palmas de Gran Canaria, experto en análisis de coste efectividad.

Expediente Nº

INVESTIGADOR/A PRINCIPAL: Josep M Montserrat

MEMORIA DE SOLICITUD PROYECTO DE INVESTIGACIÓN EN SALUD
SECCIÓN JUSTIFICACIÓN DETALLADA DE LAS PARTIDAS PRESUPUESTARIOS SOLICITADAS
(Ajústese al espacio disponible)

PERSONAL. Tecnico superior. La experiencia previa con otros ensayos clínicos evidencia la necesidad de disponer de una monitorización externa precisa al igual que en los ensayos de la industria farmacéutica. El coste total **2 años (tiempo de inclusión) : 59000** (según tabla salarial ISCIII)

BIENES Y SERVICIOS
A) : Pruebas sueño: Muchas pruebas previstas de sueño son las que harían de una forma asistencial. Sin embargo, dadas las listas de espera y nuestro ritmo, para que el protocolo se lleve a cabo en un tiempo razonable es preciso priorizar y en consecuencia hacer un número de pruebas extra que se estima sea el 50% de la muestra total. De estos estudios, tanto diagnósticos como de titulación, se estima como mínimo que un 65% requerirán poligrafía respiratoria y un 35% titulación de CPAP
El 50% de TOTAL (202): 101 estudios:
Diagnósticos: 65 poligrafías respiratorias a 100 E: 6500 E + 35 polisomnografías convencionales a 300 E: 10500 E

TOTAL DIAGNOSTICO: 17000E

Tratamiento: De un total 101 pacientes requieran titulación un 60%: 60 pacientes
25 PSG convencional a 300 E: 7500 y 35 con poligrafía respiratoria 100 &: 3500 E
TOTAL TRATAMIENTO: 11000 E

TOTAL Pruebas sueño: 28000

B): Análisis estadístico y manejo de Bases de Datos aspectos trascendentales del proyecto para valorar la posibilidad de transferencia 17500 E

C): Análisis del coste - efectividad mediante modelos bayesianos. 13900 E

D): EQUIPAMIENTO TIC's. Se dispone parte del equipamiento pero se solicita los más recientes ligados al diagnóstico para la transmisión de datos. Se disponer ya de los equipos de titulación 25000 E

E): Desarrollo e implementación pagina web .14000 E

F): Mantenimiento pagina web 2880 E

G) Primer año: Prestación de servicios para desarrollo y control rama TIC. Desarrollo TIC y ya su monitorizacion inicial**.** 16000 E

H): Segundo año y mitad año 3: Servicios de monitorización rama TIC : 17000 E

I): **Presentación a Congresos**. 4000€

REUNIONES Y VIAJES. El grupo especialista en el análisis de coste efectividad reside en Palma de Gran Canaria (Apartado F). 3500€

Expediente Nº

INVESTIGADOR/A PRINCIPAL: Josep M Montserrat

MEMORIA DE SOLICITUD PROYECTO DE INVESTIGACIÓN EN SALUD
SECCIÓN PRESUPUESTO

Presupuesto solicitado

**1. Gastos de Personal**	**Euros**
Técnico superior - (monitor, periodo inclusión 2 años)	59.000
**Subtotal Gastos de Personal :**	**59.000**

2. Gastos de Ejecución

A) Adquisición de bienes y contratación de servicios
(Bienes inventariable, material fungible y otros gastos)

A) Pruebas de sueño.	28.000
B) Anásis estadistico y manejo de BBDD	17.500
C) Análisis de coste-efectividad mediante modelos bayesianos	13.900
D)Equipamiento modelo diagnóstico transmisión telemática	25.000
E) Desarrollo e implementación de la página web	14.000
F) Mantenimineto pagina web	2.880
G) Primer año: Prestación de servicios para desarrollo y control rama TIC	16.000
H) Segundo año y mitad año 3: Servicios de monitorización rama TIC	17.000
I) Presentación a Congresos	4.000
**Subtotal Gastos Bienes y Servicios :**	**138.280**

B) Gastos de Viajes

F) Reunión grupo investigador. Grupo coste.efectividad Gran Canaria	3.500
**Subtotal Gastos Viajes :**	**3.500**
**Subtotal Gastos Ejecución :**	**141.780**
**Total Presupuesto :**	**200.780**

Expediente Nº

INVESTIGADOR/A PRINCIPAL: Josep M Montserrat

MEMORIA DE SOLICITUD PROYECTO DE INVESTIGACIÓN EN SALUD
SECCIÓN ANEXOS

INTRODUZCA TEXTO COMO ANEXO **Máximo 3 páginas.**

HOJA INFORMATIVA DEL EL ESTUDIO

Aplicabilidad, eficacia y coste efectividad de un modelo de gestión para el síndrome de apneas durante el sueño basado en telemedicina

¿Qué es la apnea del sueño?

Usted puede padecer una enfermedad que se llama síndrome de apneas del sueño, que consiste en la obstrucción o colapso de la faringe durante el sueño, ocasionándole paradas respiratorias. Esta enfermedad produce un aumento de la tendencia al sueño durante el día, así como mayor probabilidad de enfermedades cardiovasculares y accidentes de tráfico.

¿Cómo se diagnostica?

La prueba de referencia para el diagnóstico es la polisomnografía, realizada en unidades de sueño hospitalarias, se basa en la colocación de unos de bandas, (torax y abdomen) cánulas y termistor (nasales) y un sensor en el dedo para mirar el oxígeno en sangre, ademas unos electrodos en la cabeza. Todo ello con la finalidad de registrar durante el sueño diferentes parámetros cardiorrespiratorios y neurofisiológicos, lo cual hace que su realización e interpretación precisen ingreso en el hospital. Otra prueba diagnóstica utilizada habitualmente es la poligrafía respiratoria que solo registra variables respiratorias, es más simple y puede realizarse a nivel domiciliario.

Con la información derivada de estas pruebas y de los síntomas que usted presenta, así como de la presencia de otras enfermedades concomitantes, se decide el tratamiento. En los casos graves consiste en la aplicación de un dispositivo que genera aire a presión conocido como CPAP. El aire a presión se transmite del dispositivo al paciente a través de un tubo y una mascarilla colocada sobre la nariz, evitando así la obstrucción de la faringe y las paradas respiratorias. En los casos leves y moderados el tratamiento consiste en observar una serie de medidas de higiene de sueño (horarios de sueño regulares, horas de sueño suficientes, etc) y dietéticas para perder peso.

¿Por qué de nuestro estudio?

Se le ofrece la posibilidad de colaborar en este estudio en el que participarán diversos pacientes que acuden como Vd al ambulatorio para se diagnosticados del síndrome de apneas. La gran diferencia de nuestro estudio es que se va a hacer totalmente de un modo extrahospitalaria empleando técnicas de telemedicina tipo videoconferencias etc. Dicho estudio está avalado por la Sociedad Española de Neumología siendo el investigador principal el Dr. Josep M Montserrat del Hospital Clinic de Barcelona.

Este estudio consiste en evaluar si el diagnóstico y la decisión de tratamiento de los pacientes con sospecha de apnea del sueño pueden ser realizados sin que Vd acuda al hospital mediante técnica de telemedicina, video conferencias para las entrevistas ademas de los estudios a través de equipos especiales que le serían llevados a su domicilio. Dicho de otro modo queremos demostrar si todo el manejo (entrevistas, estudios etc) de pacientes como Vd es mejor y con menor coste haciéndolo en domicilio. Se pretende además valorar si Vd se sentirá más cómodo pues no precisará desplazarse. Esto implicaría que un número importante de pacientes podrían diagnosticarse y tratarse fuera del hospital.

¿Qué implica su participación?

Usted será aleatorizado para realizar el diagnóstico y la decisión del tratamiento por el procedimiento clásico hospitalario o por el procedimiento telemático. El equipo investigador le seguirá durante 6 meses realizando 4 evaluaciones, una inicial, otra intermedia al mes del comienzo del tratamiento, a los tres meses y la última a los seis meses de éste. En el periodo de seguimiento, además de las pruebas de diagnóstico comentadas, en la evaluación inicial, se le practicarán unos cuestionarios y, al inicio y final del estudio y medidas de presión arterial. Ninguna de las pruebas a realizar son peligrosas para usted ni implican ningún riesgo. Serán las mismas que se realizan en el hospital. El estudio permitirá mejorar el conocimiento de su enfermedad y elegir cuál será el proceso de diagnóstico y de decisión de tratamiento más adecuado en casos como el suyo, lo que nos ayudará a mejorar el rendimiento de este procedimiento en el futuro eligiendo el sistema más efectivo y con menor coste.

Si no desea participar

Usted **no tiene ninguna obligación de participar en el estudio**, es totalmente voluntario. Si decidiera no participar, esto no afectaría en su cuidado médico ni en las decisiones de su tratamiento. Su médico le responderá a todas aquellas preguntas que se le planteen antes y durante el transcurso del estudio. Si inicialmente aceptara su participación pero posteriormente no quisiera continuar, podría abandonarlo sin ninguna recriminación.

Seguridad y confidencialidad del paciente

Este estudio ha sido aprobado por la Comisión de Investigación del Hospital, la Comisión de Ética y Ensayos Clínicos y la Dirección del Hospital. El equipo médico estará formado por médicos de atención primaria y especialistas con amplia experiencia en esta enfermedad. Todos sus datos personales y los derivados de los pruebas realizadas y cuestionarios serán tratados de forma confidencial de acuerdo a la Ley Orgánica 15/1999, de 13 de diciembre, de Protección de Datos de Carácter Personal.

Si a lo largo del estudio tuviera cualquier duda o problema no dude en ponerse en contacto con el equipo de investigación llamando al teléfono______________________.

El Dr/Dra ___________________ me ha proporcionado una copia de la información completa acerca del estudio.

HOJA DE ACEPTACIÓN DE PARTICIPACIÓN EN EL ESTUDIO

El Dr/Dra. ___________________ me ha dado la oportunidad de preguntar sobre cualquier cuestión relacionada con el estudio. Me ha explicado que soy libre de abandonarlo en cualquier momento si lo deseara, sin que ello repercuta en el seguimiento de mi enfermedad ni en el tratamiento médico.

He leído y comprendido todo lo que se me ha explicado, y consiento en participar en el estudio bajo mi responsabilidad:

Nombre del paciente:..Firma

Nombre del investigador ....Firma..................................Fecha:

Expediente Nº

INVESTIGADOR/A PRINCIPAL: Josep M Montserrat

INTRODUZCA IMÁGENES COMO ANEXO **Máximo 1 página.**
