# Supplementary material for: Comprehensive management of obstructive sleep apnea by telemedicine: Clinical improvement and cost-effectiveness of a Virtual Sleep Unit. A randomized controlled trial
Source: PLoS One. 2019 Oct 24;14(10):e0224069. doi: 10.1371/journal.pone.0224069 (PMC6812794; doi:10.1371/journal.pone.0224069)
Supplement: S4 File — (DOCX) [file pone.0224069.s012.docx]

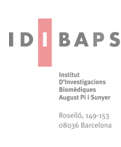


Ministry of Economy and Competitiveness

Carlos III Health Institute

S.D.G. for Evaluation and

Promotion of Research

Avda. Monforte de Lemos, 5

28029, Madrid

**Ref: Request for change in project methodology**

**File: PI14/00416**

In reference to the project with file number PI14/00416 for which I am the principal investigator, I hereby inform you of the following changes in methodology. The project "Applicability, efficiency and cost-effectiveness of a management model for SAHS based on TIC´s" contemplates the telematic management of SAHS from diagnosis to treatment. Heterogeneous patients that require a series of analysis and personalized management to avoid the bias of the different professionals involved and 2. The creation of a new fully automatic branch that fits even better in the context of the study. In order to solve these aspects, much more experience in sleep diseases is needed to give the maximum quality to the study, a professional expert in sleep medicine is needed.

**SUMMARY PROPOSED INITIAL SCHEDULE**

**First 6 months:** Implement all the necessary means: website, registration for electronic entry, meetings to define well the protocols and registration / reading of sleep analysis.

**Next 24 months** : Recruitment of patients who come to the sleep units on suspicion of SAHS and carrying out the corresponding studies mentioned in the project. It is the estimated time for the entry of all patients and their follow-up.

**Last 6 months**: Analysis of the results with the help of a statistician with experience and the first communications to congresses.

**NEW PROPOSED CRONOGRAMME** (there is a delay of 3 months as stated in the annual report)

**First 9 months** (January 2015 - November 2015) All the necessary means have already been put in place: web page, registration for electronic entry, meetings to define well the protocols and registration/reading of the different pilot tests that are already being carried out and partially analysed.

**Next 22 months** (December 2015-September 2017): According to the previous evidences the requested personnel will do the following functions: 1) select the patients adequately to randomize to avoid anomalous requests of the out-of-hospital doctors, 2. The patients randomized in any of the branches will carry out the interview and will supervise the sleep studies, 3. In the patients randomized later will carry out the video conference and finally 4. will be the consultant of the nurse in charge of the follow-up of the patients. All this for a homogenization of criteria that generate adequate results to proceed to multi-center work and transfer to the health system.

**Last 3 months** (October-2017-December 2017). During this period of time, the following will be controlled: 1. The various statistical analyses and cost effectiveness and 2. The first communications will be made to congresses.

This variation of the protocol will not lead to any increase in the execution of the project. In all its context, the scheme would be as follows

1) The contracted professional who according to the previous arguments will optimize and improve the whole study.

2) We request the transfer of 38,718.75 € from the Item Goods and Services to Personnel due to the fact that the estimated cost for the hiring of personnel who will carry out the complementary analyses from April 2016 to December 2017 is 38,718.75 € (hiring of a graduate at 30h/week, 16,593.75 € from April to December 2016 and 22,125 € from January to December 2017).

3) This transfer of the balance is possible due to the fact that the concepts of the application referring to the provision of services of the ICT branch/telematic transmission and diagnostic model equipment will be available from other sources of financing, which may lead to significant savings and allow for a considerable improvement in the execution of the project if they accept the modification of the budget that we are requesting.

And for this to be recorded, this document is signed at the place and date indicated above.

VºBº Mrs. Rosa Vilavella Gasull IDIBAPS manager

Dr. Josep María Montserrat Principal Investigator

Methodology modification. During the first 18 months the technical setup was prepared. Before the start the study design was modified in part: 3 months follow-up and non-inferiority analysis (see the manuscript submitted) .
